# A validated survey to measure household food waste

**DOI:** 10.1016/j.mex.2019.10.029

**Published:** 2019-10-31

**Authors:** Erica van Herpen, Lisanne van Geffen, Mariska Nijenhuis-de Vries, Nancy Holthuysen, Ivo van der Lans, Tom Quested

**Affiliations:** a Marketing and Consumer Behavior Group, Wageningen University, the Netherlands; b Wageningen Food and Biobased Research, the Netherlands; c WRAP, United Kingdom

**Keywords:** Household food waste questionnaire, Food waste, Questionnaire, Measurement, Consumer

## Abstract

To assess household food waste in large-scale studies with the aim to understand differences in food waste levels between households, surveys are often employed. Yet, survey measures rely on people’s awareness of their own food waste levels, draw upon their memory of instances of food waste, and can be subject to social desirability. Therefore, existing survey measures may not be optimal in measuring household food waste. The *Household Food Waste Questionnaire* has been developed to ameliorate these issues. It uses a pre-announcement to increase awareness of food waste, focuses on a short and specific time period (i.e. the past week), and specifies detailed product categories, whereas previous surveys mostly used general questions without reference to time period or product category. The amount of household food waste estimated using the *Household Food Waste Questionnaire* is likely to considerably underestimate the actual amount, so the method should not be used to obtain accurate waste amounts, but rather to distinguish differences between households and/or across time. Advantages compared to existing survey methods are that it:

• Distinguishes better between households with low versus high levels of food waste
• Correlates more highly with other measurements of household food waste (diary, kitchen caddy, photograph coding)

## Graphical abstract

**Figure:**
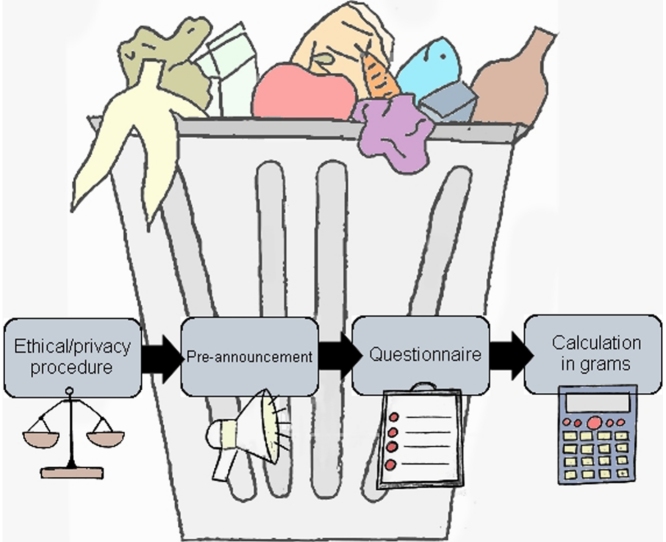

**Specification Table**

**Table:** 

Subject Area:	*Social sciences*
More specific subject area:	*Household food waste*
Method name:	*Household Food Waste Questionnaire*
Name and reference of original method:	*NA*
Resource availability:	*NA*

## Method details

Food waste has become a global concern and a priority on the political agendas [1], and especially household food waste is high in developed countries [2]. Although there is a growing body of literature on food waste quantification in the past years, there are still many issues remaining. This has led Xue et al. [1] to call for “more consistent, in-depth, and primary-data-based studies”. In response, the current article proposes the *Household Food Waste Questionnaire*, as a possible measurement method suitable for large representative samples and especially useful for studies that attempt to explain differences among households.

Various methods can measure household food waste and gain insights into why it is generated, such as diaries, waste composition analysis, and surveys. These methods all have their advantages and disadvantages, related to time, cost, accuracy, objectivity, and reliability [1], with none clearly superior to the others. Although waste composition analysis provides detailed and accurate insights into the level of food waste [3], this approach is impractical for use across large samples of households. Prior scientific research has provided suggestions for the development of other practical measures, including information technology (ICT) tools, surveys, diaries and combinations of these [[4], [5], [6]], but their validity in large scale household samples is unclear. Especially a valid survey measurement would be useful, as this can be applied at relatively low monetary costs for the researcher as well as relatively low effort for participating households. Yet, survey measures rely on people’s awareness of food waste levels, draw upon people’s memory, and can be subject to social desirability, which needs to be taken into account when developing a new survey to assess household food waste.

Results of several prior studies have shed a gloom light on the ability of surveys to provide an accurate measure of food waste amounts. In line with prior research [2], Giordano et al. [7] found that also nowadays awareness of the amount of food waste is very low, with households substantially underestimating the amount of food waste in their home, when compared to information from diaries. To diminish this lack of awareness, our questionnaire includes a pre-announcement, which aims to increase awareness of the amount wasted in the measurement period. Delley and Brunner [8] found a striking tenfold discrepancy between a survey measure based on perceived average food waste per week and extrapolations from waste composition analysis. A potential cause could be that an average week is difficult for consumers to imagine. Food waste is unplanned for, and can easily be trivialized as being due to exceptional circumstances and therefore not part of what would constitute an average week. We therefore focus our questions on a specific week (i.e. the week prior to filling in the questionnaire), to diminish this potential bias. Further, Elimelech et al. [15] found that estimates of perceived amounts of waste across product categories (on a 6-point scale ranging from ‘none’ to ‘large amount’) did not correlate well with actual amounts based on waste composition analysis. Their results for perceived proportions of food waste likewise indicated that respondents’ estimations were vastly inaccurate. We consequently decided to allow respondents to answer in everyday units that they are familiar with, that is, instead of indicating whether a little or a lot is wasted they are asked to answer in units such as pieces, slices, or spoons, for which differences in interpretation might be smaller.

Thus, the *Household Food Waste Questionnaire* has been developed to overcome issues related to self-reported survey measures of household food waste. It uses a pre-announcement to increase awareness of food waste, focuses on a short and specific time period (i.e. the past week), specifies detailed product categories, and allows respondents to provide answers in units that they are familiar with, whereas previous surveys mostly used general questions without reference to time period or product category. To keep respondent burden (time and effort) low, they are first asked whether food waste has occured in a particular category and only asked for the amount and type of food waste when their answer is positive. Keeping respondent burden low is important because prior research on measurements of household food waste has indicated a high abandon rate when the amount of work asked from respondents is high [7]. The survey has been developed and assessed by van Herpen et al. [9], and has been improved upon and translated into Dutch, German, Hungarian, and Spanish as part of the EU project REFRESH [10]. The survey relies on respondents’ memory of food waste occurring in the past week, which is a tradeoff between the advantages of using a short time interval (less reliance on memory) and a long time interval (less increase in awareness and potential behavioral change).

The following sections describe details of the procedure of the *Household Food Waste Questionnaire* and the survey questions itself. The survey is available in five languages (English, Dutch, German, Hungarian, and Spanish) in the appendices. When using the survey for households in different countries, we strongly recommend translating the questions into the national language, and paying particular attention to providing relevant examples for the product categories and measurement units.

## Procedure

The procedure consists of the following elements:

1 Ethical/privacy check
2 Pre-announcement
3 Questionnaire
4 Calculation of household food waste in grams from the reported units

### Ethical/privacy procedures

The first step is to check ethical and/or privacy procedures that need to be set in place before the implementation of the study. This involves regulation at the institution, national, and international (e.g., EU) level. Please be aware that personal information of the respondents will be gathered during the research. The ethical/privacy procedures usually involve gaining ethical clearance from an ethical committee, providing respondents with a detailed consent form prior to the study to obtain informed consent, and removing any information that can be related back to an individual from the dataset (anonymizing the data).

### Pre-announcement

Most consumers are not very aware of the amount of food they are wasting at home [2,7]. Therefore, questioning them on their amount of food waste without a prior ‘heads up’ can lead to unreliable data. To mitigate this effect, a pre-announcement to the respondents can make food waste more salient and will consequently lead to more reliable data. Please be aware that although this method increases the validity of the measurement data, it also increases the awareness of the respondents on their waste behavior. Thus, when this measurement methodology is used in combination with an investigation into respondents’ current level of awareness, such awareness questions should be asked beforehand. Moreover, increased awareness due to the pre-announcement could have a potential influence on actual food waste behavior, even when emphasizing that respondents should try not to change their behavior during the study. Given that underestimation due to unawareness appears to be a substantial issue (see e.g., [8]), we advocate the use of the pre-announcement despite its potential effects on social desirability or behavior change. The questionnaire concerns food waste in the past week and is hence sent one week after the pre-announcement.

Example text of the pre-announcement:

**Table:** 

Welcome to this research and thank you for participating! In this research we would like to know how food is handled in your household. For this study, we would like to ask you to pay close attention to the food and drink products that are thrown away in your household in the upcoming week: from [DATE] up to and including [DATE]. After this week, you will receive a survey with questions about what has been thrown away. This survey will be about all edible food and drink products you have bought in the (online) (super) market or have home-grown, that are thrown away. This also includes products that are spoiled or past their expiration date. It does not matter if you have thrown the food away in the general trashcan, food waste container, compost heap or gave it to an animal (pet, birds, et cetera), or otherwise. It is all included. It will not be about bones, peels, seeds or stumps, or food and drink products that are thrown away when eating in a restaurant or canteen. Thank you very much in advance!

When social desirability is a concern, attempts can be made to diminish this through the way in which the questionnaire is introduced in the pre-announcement. By describing food waste in neutral terms, as food that is brought into the household but not consumed, some of the negativity surrounding food waste could be reduced. Other ways to diminish social desirability could be to emphasize the anonymity of respondents, the high occurrence of food waste, and/or the research need for accurate answers.

### Questionnaire

The questionnaire contains questions on the respondents’ food waste amounts in the past week, specified over various food product categories and four food waste states which are connected with stages of the consumption life cycle in which consumers discard their food (planning, shopping, storing, preparing, and consuming; [11]). “Food”, for the purpose of this questionnaire, is defined as edible fractions of food products that are intended to be eaten by humans. This excludes inedible fractions, such as bones, peels, seeds, stumps, etc. Selection of food categories was based on prior research [12,13]. Originally, 22 categories were distinguished, and later this was updated to 24 food categories. For future research, we advise to make “Milk” a separate category, bringing the number of categories to 25, as also indicated in the appendices with the questionnaires.

A pilot study among 30 respondents, who were recruited from the consumer panel of Wageningen Food and Biobased Research, tested an initial version of the questionnaire. In this version, respondents indicated how often various foods were eaten in the household and the proportion of the food that is wasted, for each of the waste stages. Respondents also rated whether questions were clear and whether they had doubts about the correctness of their answer (on 100-point slider scales). Open-ended questions were included throughout the questionnaire to allow respondents to freely give their opinion about the instructions and questions.

Results of the pilot study indicated no problems with the selection and delineation of waste stages. No missing stage was reported, and although several respondents indicated that understanding the waste stages was not always easy, they indicated that they were able to use them. Some respondents suggested that examples of the specific food category in each of the waste stages would be helpful, and we took up this suggestion in the final questionnaire.

Results did indicate missing and unclear food categories, which made us adjust the product category list. Respondents also indicated that the category scale used to measure food waste, with categories for different proportions, was difficult to use. A further issue with this scale is that it does not measure absolute levels. For these reasons, we decided to adjust the measurement scale and to use absolute amounts instead. The final questionnaire uses everyday units (e.g., spoons, pieces, slices, liters) to express the amount of food waste in units that respondents are familiar with. These can be easily transferred into grams, to be in line with other measurements and policy targets, and potentially also into other units (e.g., calories).

The final questionnaire consists of the following parts:

• General introduction, in which food waste is defined and explained.
• A list of categories; respondents are asked to tick the boxed of the products that are disposed of in their household in the past week (multiple answers possible). This question is answered by all respondents.
• An explanation of food waste states.
• Follow-up questions for those food categories that were ticked only. These concern (a) how much was disposed of in the past week, and (b) in which state was the majority of the disposed of food. Amounts are asked in units that are appropriate for the product category, so as to obtain reliable estimates. For each question, five answer options are provided, and in the updated *Household Food Waste Questionnaire* reference amounts are provided to help respondents make accurate estimates. States are described with examples from the product category. In the surveys in different languages, these examples are tailored to the food context of each country and are included to help respondents understand what the different food waste states entail.

An example text for the explanation of food waste states is:

**Table:** 

We split food waste into several categories, which are explained below. Please read this carefully as these categories will be used in the next questions Food waste can be categorized into: 1) Completely unused foods: food that is disposed of which has not been used at all. For instance, unopened packages, including unopened parts of multipacks, moulted apples, dried leek, complete bread. 2) Partly used foods: food that is disposed of after it has been party used. For instance, a few bread slices, halve a package of meat cuts, halve an onion or halve a package of milk. 3) Meal leftovers: leftovers that are disposed of after these were left on the plate, pots or pans. For instance, potato mash or rice that is left on the plate or in the pan, sandwiches that were not eaten during lunch. 4) Leftovers after storing: meal leftovers that are disposed of after these were stored in the fridge or freezer to be eaten at a later moment. For instance, a frozen pasta portion of last week. You will receive several questions about different type of food and drink products you have disposed of in the past week. First, we ask how much of a certain product your household disposed of in the past week. Next, we ask to which category (unused, partly used, meal leftovers, leftover after it was stored) the majority of the disposed of food product belonged when it was disposed of. Please pay attention to which food product it refers!

An example of the follow-up questions is:

**Table:** 

Fresh vegetables and salads. In your household, how much fresh vegetables and salads was disposed of in the past week? *One serving spoon equals 50 gram. As a reference: this equals half a leek or four mushrooms.* • Less than one serving spoon • 1 to 2 serving spoons • 3 to 4 serving spoons • 5 to 6 serving spoons • More than 6 serving spoons To which category did the (majority) of the disposed of fresh vegetables and salads belong? *Please tick the category that occurred the most. You can tick more than one box if multiple categories occurred in the same amount.* □ Completely unused foods: Food that is disposed of while it has not been used at all (e.g., a leek) □ Partly used foods: Food that is disposed of after it is partly used (e.g., half an union) □ Meal leftovers: Meal leftovers that are disposed of after these were left on the plate, pots or pans □ Leftovers after storing: Meal leftovers that are disposed of after these were stored

It is important to note that the follow-up questions are only asked for those product categories for which it was indicated that food was wasted in the past week. Thus, although the *Household Food Waste Questionnaire* may seem extensive, this procedure ensures that respondent burden is kept at a low level. The questionnaire is especially suitable for online administration, as the questions that are not relevant can be programmed to not appear. Furthermore, the questions on food waste state are only relevant when this level of detail is needed for the research at hand, and can potentially be skipped.

The questionnaire is detailed in the appendices:

• Appendix A Questionnaire in English
• Appendix B Questionnaire in Dutch
• Appendix C Questionnaire in German
• Appendix D Questionnaire in Hungarian
• Appendix E Questionnaire in Spanish

Questions in the appendices are numbered for easy reference, but should be shown unnumbered to respondents, to prevent confusion due to skipping of questions.

### Calculation of household food waste in grams from the reported units

As the units for the reported amount of food waste differ across the product categories (e.g., spoons, pieces, slices, liters), these need to be recalculated into a common measurement unit to obtain a measure for total household food waste. To be comparable with prior research, this common unit is preferably grams.

Appendix F provides information on the assumed amount of grams per unit, which can be used for calculation, as well as the specific values that were used based on the answer options in the questionnaire. For answer options in which a range is provided (e.g., 1–2 serving spoons), the mean value is used in the calculation (in this case, 1.5 serving spoons). For open-ended answer options (e.g., more than 6 serving spoons) one unit is added to the provided number in the calculation (in this case, 7 serving spoons). The amount of food waste per state is calculated by dividing the amount of food waste of each category by the number of states ticked by the respondent. Thus, if a respondent for example reports 100 g of vegetable food waste and ticks the unused and leftovers boxes, then the assumption is that the respondent has wasted 50 g of unused and 50 g of leftovers vegetables.

Summarizing the recalculated amounts per food product category lead to the estimation of the total amount of food waste per household per week. It is possible to also examine the amount of food waste (in grams) for each of the categories separately, or for overarching categories (e.g., grams of wasted vegetables by summing across fresh and non-fresh vegetables).

Please note that food waste amount is on household level, not per capita. For research looking into per capita household food waste amounts, the number of persons that joined for dinner during the measuring week should be taken into account.

## Sample selection

Studies using the food waste questionnaire to assess the amount of household food waste, should take care when selecting samples. Samples should be sufficiently large and representative. As household demographics, such as household size, have been shown to affect the amount of household food waste, we recommend setting quotas on them whenever the results need to be representative for a target population. These quotas should be set at the household level. For instance with regard to household size, quotas should be calculated by considering population figures about the number of households of a particular size in the country (and not the number of inhabitants living in households of a particular size).

## Method validation

To assess the validity of the questionnaire, a comparison can be made with other measurements of household food waste. These other measurements could be other surveys, but also measures such as diaries, waste-composition analysis, self-collection in kitchen caddies, in-home observations, or photographs. As food waste differs greatly across households, exposing households to different measurements in a between-subject design, and comparing mean food waste across measurements, would necessitate very large samples. A within-subject design in which households provide information on food waste using different measurements at the same point in time should provide more reliable estimates, also for relatively small samples.

Repeated-measures ANOVA, with measurement as within-subjects factor, can test the validity of the mean food waste amount when compared to other measurements. In addition, the agreement between measured individual-household level food waste amounts by the questionnaire and those from other measurements can be assessed by differences and by measures of association such as the Pearson correlation (with interval-scaled measures), Kendall’s tau (with ordinal-scaled measures), and Tucker’s congruence coefficient (with ratio-scaled measures).

In setting up such a validation study, care should be taken as the measurements themselves could influence each other. Especially surveys, including the questionnaire advocated in the current article, are prone to disruption due to other measurements as these other measurements increase awareness of food waste and, as a consequence, could influence memory and thus responses to the surveys. This can be taken into account in the procedure by administering the survey measurement first, or by using unobtrusive measurements (e.g., waste composition analysis of waste put at the curb).

An example of a way to test whether some other measurement (O) has an impact on the responses to our questionnaire (Q) (without being hampered by large differences between households) would be to measure food waste at two points in time, in two experimental groups. In one group food waste in a first time period is only measured by our questionnaire (Q), and in the a second time period measured by both the other measurement and our questionnaire (O + Q). Food waste in the other group is measured by both the other measurement and our questionnaire (O + Q) for both time periods. The differences in reported food waste and correspondence between the weeks as measured by our questionnaire, can then be compared across both groups, to assess if the other measurement (O) influenced responses to our questionnaire (Q).

Another requirement for the questionnaire is that it is able to distinguish between households with higher and lower food waste. Such discriminatory power can be assessed by such measures of variation as the range, the standard deviation (for interval-scaled measures), the coefficient of variation (for ratio-scaled measures) or the interquartile range (for ordinal measures).

The survey method described in this article has been assessed and compared to other measurements of household food waste, using an approach similar to what is described above, by Van Herpen et al. [9]. This study took place among 143 members of a consumer panel, in which respondents provided information at two points in time, using various measurement approaches. Results show that the measure obtained from the *Household Food Waste Questionnaire* correlates highly with other measurement approaches (diary, kitchen caddy, photo coding).

Van Herpen et al. [9] have also compared the food waste questionnaire to other survey measures. The following survey measures were used as comparison: a general question on how much food is wasted (answered on a category scale from “Quite a lot” to “None”), a relative question on the percentage of food that is bought that is wasted (answers ranging from “None” to “More than 50%”), the frequency with which food is wasted (answers on a category scale ranging from “Regularly” to “Never”), and the proportion of food wasted in different product categories (answers ranging from “Nothing” to “More than half”). Results of the study show that these measures are less valid, as they lead to large underestimation of the level of food waste, low variability in reported food waste across households compared to other methods, and low correlations with other measures. The *Household Food Waste Questionnaire* clearly outperformed them on each of these points.

Despite these clear advantages, the *Household Food Waste Questionnaire* appeared to significantly underestimate the amount wasted. The average weight of the household food waste per week was 639 g according to the *Household Food Waste Questionnaire*, compared to estimates of 1122 g based on diary entries, 1042 g based on kitchen caddies, and 1208 g when information from both diaries and kitchen caddies was combined. Although the difference is large, with the questionnaire accounting for only 61% of the food waste recorded in the waste collection through kitchen caddies, this is substantially more accurate than what was found in previous studies [8,14]. Specifically, Delley and Brunner [8] found a tenfold discrepancy between a survey based on perceived average food waste per week and extrapolation from waste composition analysis.

Future research could further validate the proposed questionnaire, and also test variations. For instance, by comparing the proposed questionnaire to waste-composition analysis, by examining which kind of pre-announcement gives the best results, or by testing different time intervals (e.g., daily versus weekly measurement of food waste). Assessing the reliability and validity of the questionnaire when applied multiple times to the same household is also a fruitful direction for future research, as this would provide insights into the usefulness of the questionnaire for pre/post-test studies on the effectiveness of interventions.

## Conclusion

In summary, the proposed *Household Food Waste Questionnaire* appears to be a useful method for large-scale measurements to differentiate households according to the amount of food waste each produces, although it should be noted that it underestimates the amount of food waste. The proposed questionnaire is especially useful when attempting to measure household food waste in large and geographically dispersed samples, and when respondent burden needs to be kept low. We advise further testing of its validity when the questionnaire is to be used to measure the effectiveness of interventions.

## Declaration of Competing Interest

The authors declare that they have no known competing financial interests or personal relationships that could have appeared to influence the work reported in this paper.
