# Supplementary material for: A validated survey to measure household food waste
Source: MethodsX. 2019 Oct 31;6:2767–75. doi: 10.1016/j.mex.2019.10.029 (PMC6889683; doi:10.1016/j.mex.2019.10.029)
Supplement: Supplementary file 1 [file mmc1.pdf]

Appendix A: Household Food Waste Questionnaire in English

Introduction

Last week you received an email to pay close attention to the food and drink products you have thrown away. This questionnaire will be about those products. As a reminder:

This questionnaire **will** be about:

- All edible food and drink products you have bought in the (online) (super) market or have home-grown that are thrown away.
- This also includes products that are spoiled or past their expiration date.
- It does not matter if you have thrown the food away in the general trashcan, food waste container, compost heap or gave it to an animal (pet, birds, et cetera), or otherwise. It is all included.

It will not be about:

- Bones, peels, seeds, or stumps.
- Food and drink products that are thrown away when eating in a restaurant or canteen.

Questionnaire

Question 1: Please tick the boxes of the products that are disposed of in your household in the past week. In case of complete meals, please report the main ingredients separately.

☐ Fresh vegetables and salads
☐ Non-fresh vegetables (jar / canned / frozen)
☐ Fresh fruit
☐ Non-fresh fruit (jar / canned / dried / frozen)
☐ Potatoes
☐ Potato products (fries, chips, baby or precooked potatoes, et cetera)
☐ Pasta
☐ Rice and remaining grains (including wraps, couscous, et cetera)
☐ Beans, lentils, chickpeas, et cetera.
☐ Meat (please report cold meat slices at “bread toppings”)
☐ Meat substitute
☐ Fish
☐ Bread toppings (cold meats slices, cheese slices, sweet topping, et cetera)
☐ Bread
☐ Cereals (muesli, granola, oat, brinta, et cetera)
☐ Yoghurt, custard, et cetera
☐ Cheese (cheese cubes, French cheese, sprinkle cheese. Excluded: cheese as bread topping)
☐ Eggs
☐ Soups / curry
☐ Sauce (ketchup, mayonnaise, cocktail sauce, et cetera)
☐ Candy / cookies / granola bars / chocolate bars
☐ Crisps / nuts
☐ Non-alcoholic beverages (milk*, juice, soda. Excluded: water, tea, coffee, diluted syrup)
☐ Alcoholic beverages
☐ I have not thrown away any food or drink products

* *NOTE*: Due to progressive insights, creating a separate category for “Milk” can be considered, as this is a frequently disposed of food product. However, please keep in mind that this will change the set-up slightly, when comparing the results with the earlier applications of this survey.

Introduction to the next part of the questionnaire

Food waste states
We split food waste into several categories, which are explained below. Please read this carefully as these categories will be used in the next questions.

1) Completely unused foods: food that is disposed of which has not been used at all. For instance, unopened packages, including unopened parts of multipacks, moulted apples, dried leek, complete bread.
2) Partly used foods: food that is disposed of after it has been party used. For instance, a few bread slices, half a package of meat cuts, half an onion or half a package of milk.
3) Meal leftovers: leftovers that are disposed of after these were left on the plate, pots or pans. For instance, potato mash or rice that is left on the plate or in the pan, sandwiches that were not eaten during lunch.
4) Leftovers after storing: meal leftovers that are disposed of after these were stored in the fridge or freezer to be eaten at a later moment. For instance, a frozen pasta portion.

You will receive several questions about different type of food and drink products you have disposed of in the past week. First, we ask how much of a certain product your household disposed of in the past week. Next, we ask to which category (unused, partly used, meal leftovers, leftover after it was stored) the majority of the disposed of food product belonged when it was disposed of. Please pay attention to which food product it refers!

NOTE: The next questions are only shown if respondents indicated waste in the respective category in Question 1

Question 2: Fresh vegetables and salads.
In your household, how much fresh vegetables and salads were disposed of in the past week?
One serving spoon equals 50 gram. As a reference: this equals half a leek or four mushrooms.
- Less than one serving spoon
- 1 to 2 serving spoons
- 3 to 4 serving spoons
- 5 to 6 serving spoons
- More than 6 serving spoons

Question 3: To which category did the (majority) of the disposed of fresh vegetables and salads belong?
Please tick the category that occurred the most. You can tick more than one box if multiple categories occurred in the same amount.
- ☐ Completely unused foods: food that is disposed of which is not used at all (e.g., a leek)
- ☐ Partly used foods: food that is disposed of after it is party used (e.g., half an union)
- ☐ Meal leftovers: meal leftovers that are disposed of after these were left on the plate, pots or pans
- ☐ Leftovers after storing: Meal leftovers that are disposed of after these were stored

Question 4: Non-fresh vegetables (jar / canned / frozen).
In your household, how many non-fresh vegetables (jar / canned / frozen) were disposed of in the past week?
One serving spoon equals 50 gram. As a reference: this equals half a leak or four mushrooms.
- Less than one serving spoon
- 1 to 2 serving spoons
- 3 to 4 serving spoons
- 5 to 6 serving spoons
- More than 6 serving spoons

Question 5: To which category did the (majority of) disposed of non-fresh vegetables belong?
Please tick the category that occurred the most. You can tick more than one box if multiple categories occurred in the same amount.
- ☐ Completely unused foods: food that is disposed of which is not used at all (e.g., unopened frozen / canned spinach package)
- ☐ Partly used foods: food that is disposed of after it is party used (e.g., half used frozen / canned spinach package)
- ☐ Meal leftovers: meal leftovers that are disposed of after these were left on the plate, pots or pans
- ☐ Leftovers after storing: Meal leftovers that are disposed of after these were stored

Question 6: Fresh fruit.
In your household, how many fresh fruits were disposed of in the past week?
One apple or banana is one piece of fruit. In case of small fruits, such as strawberries or grapes, one small bowl is considered 'one piece'.
- Approximately one fourth of a piece of fruit or less
- Approximately half a piece of fruit
- Approximately 1 piece of fruit
- 2 to 4 pieces of fruit
- More than 4 pieces of fruit

Question 7: To which category did the (majority of) disposed of fresh fruit belong?
Please tick the category that occurred the most. You can tick more than one box if multiple categories occurred in the same amount.

- ☐ Completely unused foods: food that is disposed of which is not used at all (e.g., an apple)
- ☐ Partly used foods: food that is disposed of after it is party used (e.g., half an apple that is not used in a dish)
- ☐ Meal leftovers: meal leftovers that are disposed of after these were left on the plate, pots or pans (e.g., half eaten apple or a fruit salad)
- ☐ Leftovers after storing: Meal leftovers that are disposed of after these were stored (e.g. fruit salad after it was stored)

Question 8: Non-fresh fruit (jar / canned / dried / frozen).
In your household, how many non-fresh fruits (jar / canned / dried / frozen) were disposed of in the past week?
One pear or peach from a can is one piece of fruit. In case of small fruits, as blueberries or tangerine wedges, one small bowl is considered 'one piece'.

- o Approximately one fourth of a piece of fruit or less
- o Approximately half a piece of fruit
- o Approximately 1 piece of fruit
- o 2 to 4 pieces of fruit
- o More than 4 pieces of fruit

Question 9: To which category did the (majority of) disposed of non-fresh fruit belong?
Please tick the category that occurred the most. You can tick more than one box if multiple categories occurred in the same amount.

- ☐ Completely unused foods: food that is disposed of which is not used at all (e.g., unopened fruit can)
- ☐ Partly used foods: food that is disposed of after it is party used (e.g., half full fruit can)
- ☐ Meal leftovers: meal leftovers that are disposed of after these were left on the plate, pots or pans (e.g. bowl with fruit)
- ☐ Leftovers after storing: Meal leftovers that are disposed of after these were stored (e.g. fruit salad after it was stored)

Question 10: Potatoes
In your household, how many potatoes were disposed of in the past week?
One serving spoon equals 50 gram. As a reference: this equals half a midsize potato.

- o Less than one serving spoon
- o 1 to 2 serving spoons
- o 3 to 4 serving spoons
- o 5 to 6 serving spoons
- o More than 6 serving spoons

Question 11: To which category did the (majority of) disposed of potatoes belong?
Please tick the category that occurred the most. You can tick more than one box if multiple categories occurred in the same amount.

- ☐ Completely unused foods: food that is disposed of which is not used at all (e.g., complete potato package)
- ☐ Partly used foods: food that is disposed of after it is party used (e.g., half a potato package)
- ☐ Meal leftovers: meal leftovers that are disposed of after these were left on the plate, pots or pans (e.g. smashed potato)
- ☐ Leftovers after storing: meal leftovers that are disposed of after these were stored (e.g. smashed potato after it was stored)

Question 12: Potato products (fries, baby potatoes, precooked potatoes, et cetera).
In your household, how many potato products (fries, precooked potatoes, et cetera) were disposed of in the past week?

- o Less than 10 fries / baby potatoes / pieces
- o 10 to 25 fries / baby potatoes / pieces
- o More than 25 fries / baby potatoes / pieces (approximately half a package of 500 gram)
- o Full package (750 gram) fries / baby potatoes / pieces
- o More than a package (750 gram) fries / baby potatoes / pieces

Question 13: To which category did the (majority of) disposed of potato products belong?
Please tick the category that occurred the most. You can tick more than one box if multiple categories occurred in the same amount.
- Completely unused foods: food that is disposed of which is not used at all (e.g., complete potato fries package)
- Partly used foods: food that is disposed of after it is party used (e.g., half a potato fries package)
- Meal leftovers: meal leftovers that are disposed of after these were left on the plate, pots or pans
- Leftovers after storing: meal leftovers that are disposed of after these were stored

Question 14: Pasta
In your household, how much pasta was disposed of in the past week?
One serving spoon equals 50 gram.
- Less than one serving spoon
- 1 to 2 serving spoons
- 3 to 4 serving spoons
- 5 to 6 serving spoons
- More than 6 serving spoons

Question 15: To which category did the (majority of) disposed of pasta belong?
Please tick the category that occurred the most. You can tick more than one box if multiple categories occurred in the same amount.
- Completely unused foods: food that is disposed of which is not used at all (e.g., complete pasta package)
- Partly used foods: food that is disposed of after it is party used (e.g., half pasta package)
- Meal leftovers: meal leftovers that are disposed of after these were left on the plate, pots or pans
- Leftovers after storing: meal leftovers that are disposed of after these were stored

Question 16: Rice and remaining grains (including wraps, couscous, et cetera).
In your household, how much rice and remaining grains (including wraps, couscous, et cetera) was disposed of in the past week?
One serving spoon equals 50 gram.
- Less than one serving spoon
- 1 to 2 serving spoons
- 3 to 4 serving spoons
- 5 to 6 serving spoons
- More than 6 serving spoons

Question 17: To which category did the (majority of) disposed of rice belong?
Please tick the category that occurred the most. You can tick more than one box if multiple categories occurred in the same amount.
- Completely unused foods: food that is disposed of which is not used at all (e.g., complete rice package)
- Partly used foods: food that is disposed of after it is party used (e.g., half rice package)
- Meal leftovers: meal leftovers that are disposed of after it was left on the plate, pots or pans
- Leftovers after storing: meal leftovers that are disposed of after it was stored

Question 18: Beans, lentils, chickpeas, et cetera.
In your household, how much beans, lentils, chickpeas, et cetera were disposed of in the past week?
- Less than one serving spoon
- 1 to 2 serving spoons
- 3 to 4 serving spoons
- 5 to 6 serving spoons
- More than 6 serving spoons

Question 19: To which category did the (majority of) disposed of beans, lentils, chickpeas, et cetera belong?
Please tick the category that occurred the most. You can tick more than one box if multiple categories occurred in the same amount.
- Completely unused foods: food that is disposed of which is not used at all (e.g., unopened bean jar)
- Partly used foods: food that is disposed of after it is party used (e.g., half full bean jar)
- Meal leftovers: meal leftovers that are disposed of after it was left on the plate, pots or pans
- Leftovers after storing: meal leftovers that are disposed of after it was stored

Question 20: Meat.
In your household, how much meat was disposed of in the past week?
A portion refers to one chicken breast, one steak, et cetera. In case of smaller pieces, as minced meat, try to estimate it in whole pieces of meat (e.g., one package of minced meat equals two portions).
- Approximately half a portion or less
- Approximately one portion
- 2 to 3 portions
- 4 to 5 portions
- More than 5 portions

Question 21: To which category did the (majority of) disposed of meat belong?
Please tick the category that occurred the most. You can tick more than one box if multiple categories occurred in the same amount.
- ☐ Completely unused foods: food that is disposed of which is not used at all (e.g., sausage package)
- ☐ Partly used foods: food that is disposed of after it is party used (e.g., half a sausage package)
- ☐ Meal leftovers: meal leftovers that are disposed of after these were left on the plate, pots or pans
- ☐ Leftovers after storing: meal leftovers that are disposed of after these were stored

Question 22: Meat substitutes.
In your household, how much meat substitutes were disposed of in the past week?
A portion refers to a vegetarian burger, et cetera. In case of smaller pieces, as minced meat, try to estimate it in whole pieces of meat (e.g., one package of minced vegetarian meat equals two portions).
- Approximately half a portion or less
- Approximately one portion
- 2 to 3 portions
- 4 to 5 portions
- More than 5 portions

Question 23: To which category did the (majority of) disposed of meat substitutes belong?
Please tick the category that occurred the most. You can tick more than one box if multiple categories occurred in the same amount.
- ☐ Completely unused foods: food that is disposed of which is not used at all (e.g., vegetarian burger package)
- ☐ Partly used foods: food that is disposed of after it is party used (e.g., half a vegetarian burger package)
- ☐ Meal leftovers: meal leftovers that are disposed of after these were left on the plate, pots or pans
- ☐ Leftovers after storing: meal leftovers that are disposed of after these were stored

Question 24: Fish.
In your household, how much fish was disposed of in the past week?
A portion refers to one fish filled, one piece of salmon, et cetera.
- Approximately half a portion or less
- Approximately a complete portion
- 2 to 3 portions
- 4 to 5 portions
- More than 5 portions

Question 25: To which category did the (majority of) disposed of fish belong?
Please tick the category that occurred the most. You can tick more than one box if multiple categories occurred in the same amount.
- ☐ Completely unused foods: food that is disposed of which is not used at all (e.g., complete fish package)
- ☐ Partly used foods: food that is disposed of after it is party used (e.g., half a fish package)
- ☐ Meal leftovers: meal leftovers that are disposed of after these were left on the plate, pots or pans
- ☐ Leftovers after storing: meal leftovers that are disposed of after these were stored

Question 26: Bread toppings (cold meats slices, cheese slices, sweet topping, et cetera).
In your household, how much bread toppings (cold meats slices, cheese slices, sweet topping, et cetera) were disposed of in the past week?
One portion is what is used on one slice of bread / sandwich / portion of baguette.
- Approximately half a portion or less
- Approximately a complete portion
- 2 to 3 portions
- 4 to 5 portions
- More than 5 portions

Question 27: To which category did the (majority of) disposed of bread toppings belong?
Please tick the category that occurred the most. You can tick more than one box if multiple categories occurred in the same amount.
- Completely unused foods: food that is disposed of which is not used at all (e.g., complete package with meat slices)
- Partly used foods: food that is disposed of after it is party used (e.g., half a package with meat slices)
- Meal leftovers: meal leftovers that are disposed of after these were left on the plate, pots or pans
- Leftovers after storing: meal leftovers that are disposed of after these were stored

Question 28: Bread.
In your household, how much bread was disposed of in the past week?
A (raisin) bun, portion of baguette or sandwich is similar to one slice of bread.
- Less than one slice of bread
- One or a few slices of bread
- Approximately half a loaf
- Approximately one loaf
- More than one loaf

Question 29: To which category did the (majority of) disposed of bread belong?
Please tick the category that occurred the most. You can tick more than one box if multiple categories occurred in the same amount.
- Completely unused foods: food that is disposed of which is not used at all (e.g., whole loaf)
- Partly used foods: food that is disposed of after it is party used (e.g., slices of bread)
- Meal leftovers: meal leftovers that are disposed of after these were left on the plate, pots or pans (e.g., bread crusts)
- Leftovers after storing: meal leftovers that are disposed of after these were stored

Question 30: Cereal (muesli, granola, oat, porridge, et cetera).
In your household, how much cereal (muesli, granola, oat, porridge, et cetera) was disposed of in the past week?
A portion is the amount of cereals used for one bowl of breakfast.
- Less than half a portion
- A half to one and a half portion
- Multiple portions (approximately half a package)
- Approximately a complete package
- Multiple packages

Question 31: To which category did the (majority of) disposed of cereals belong?
Please tick the category that occurred the most. You can tick more than one box if multiple categories occurred in the same amount.
- Completely unused foods: food that is disposed of which is not used at all (e.g., complete cereal package)
- Partly used foods: food that is disposed of after it is party used (e.g., half a cereal package)
- Meal leftovers: meal leftovers that are disposed of after these were left on the plate, pots or pans
- Leftovers after storing: meal leftovers that are disposed of after these were stored

Question 32: Yoghurt, custard, et cetera.
In your household, how much yoghurt, custard, et cetera was disposed of in the past week?
A portion is a small bowl with yoghurt / custard / et cetera.
- Less than half a portion
- A half to one and a half portion
- Multiple portions (approximately half a litre package)
- Approximately a complete litre package
- Multiple litre packages

Question 33: To which category did the (majority of) disposed of yoghurt, custard, et cetera belong?
Please tick the category that occurred the most. You can tick more than one box if multiple categories occurred in the same amount.

- ☐ Completely unused foods: food that is disposed of which is not used at all (e.g., complete yoghurt package)
- ☐ Partly used foods: food that is disposed of after it is party used (e.g., half a yoghurt package)
- ☐ Meal leftovers: meal leftovers that are disposed of after these were left on the plate, pots or pans
- ☐ Leftovers after storing: meal leftovers that are disposed of after these were stored

Question 34: Cheese (cheese dices, French cheese, sprinkle cheese; excluded: cheese as bread topping).
In your household, how much cheese (cheese dices, French cheese, sprinkle cheese; excluded: cheese as bread topping) was disposed of in the past week?
A handful of cheese can be seen as a dice of cheese.

- o Less than one dice of cheese
- o Approximately one dice of cheese
- o 1 to 3 cheese dices
- o 4 to 5 cheese dices
- o More than 5 cheese dices

NOTE with Question 34: Due to progressive insights, increasing the measure options can be considered, as 5 cheese dices is low compared to the potential maximum gram of waste of the other categories. However, please keep in mind that this will change the set-up slightly, when comparing the results with the earlier applications of this survey.

Question 35: To which category did the (majority of) disposed of cheese belong? *Please tick the category that occurred the most.*
You can tick more than one box if multiple categories occurred in the same amount.

- ☐ Completely unused foods: food that is disposed of which is not used at all (e.g., complete French cheese)
- ☐ Partly used foods: food that is disposed of after it is party used (e.g., partly used French cheese)
- ☐ Meal leftovers: meal leftovers that are disposed of after these were left on the plate, pots or pans
- ☐ Leftovers after storing: meal leftovers that are disposed of after these were stored

Question 36: Eggs.
In your household, how many eggs were disposed of in the past week?

- o Less than 1 egg
- o 1 egg
- o 2 to 3 eggs
- o 4 to 5 eggs
- o More than 5 eggs

Question 37: To which category did the (majority of) disposed of eggs belong?
Please tick the category that occurred the most. You can tick more than one box if multiple categories occurred in the same amount.

- ☐ Completely unused foods: food that is disposed of which is not used at all (e.g., complete eggs)
- ☐ Partly used foods: food that is disposed of after it is party used (e.g., egg white)
- ☐ Meal leftovers: meal leftovers that are disposed of after these were left on the plate, pots or pans
- ☐ Leftovers after storing: meal leftovers that are disposed of after these were stored

Question 38: Soups / curry.
In the household, how much soup / curry was disposed of in the past week?

- o Less than half a ladle
- o Half to one and a half ladle
- o Multiple ladles (approximately half a litre)
- o Approximately 1 litre
- o More than 1 litre

Question 39: To which category did the (majority of) disposed of soup belong?
Please tick the category that occurred the most. You can tick more than one box if multiple categories occurred in the same amount.

- ☐ Completely unused foods: food that is disposed of which is not used at all (e.g., complete soup package). Not applicable in case of home-made soup
- ☐ Partly used foods: food that is disposed of after it is party used (e.g., half a soup package). Not applicable in case of home-made soup
- ☐ Meal leftovers: meal leftovers that are disposed of after these were left on the plate, pots or pans (warmed package of soup or home-made soup)
- ☐ Leftovers after storing: meal leftovers that are disposed of after these were stored

Question 40: Sauces (ketchup, mayonnaise, cocktail sauce, et cetera).
In your household, how much sauce (ketchup, mayonnaise, cocktail sauce, et cetera) was disposed of in the past week?
One tablespoon equals 15 grams.
- o Less than a table spoon
- o 1 to 3 table spoons
- o Multiple table spoons
- o Approximately half a jar / bottle
- o More than one jar / bottle

Question 41: To which category did the (majority of) disposed of sauces belong?
Please tick the category that occurred the most. You can tick more than one box if multiple categories occurred in the same amount.
- ☐ Completely unused foods: food that is disposed of which is not used at all (e.g., complete sauce jar)
- ☐ Partly used foods: food that is disposed of after it is party used (e.g., half a sauce jar)
- ☐ Meal leftovers: meal leftovers that are disposed of after these were left on the plate, pots or pans
- ☐ Leftovers after storing: meal leftovers that are disposed of after these were stored

Question 42: Candy / cookies / granola bars / chocolate bars.
In your household, how much candy / cookies / granola bars / chocolate bars were disposed of in the past week?
A portion is a handful of sweets, small chocolate bar, a cookie, et cetera.
- o Approximately half a portion or less
- o Approximately one portion
- o 2 to 3 portions
- o 4 to 5 portions
- o More than 5 portions

Question 43: To which category did the (majority of) disposed of candy belong?
Please tick the category that occurred the most. You can tick more than one box if multiple categories occurred in the same amount.
- ☐ Completely unused foods: food that is disposed of which is not used at all (e.g., one cookie package)
- ☐ Partly used foods: food that is disposed of after it is party used (e.g., half a cookie package)
- ☐ Meal leftovers: meal leftovers that are disposed of after these were left on the plate, pots or pans
- ☐ Leftovers after storing: meal leftovers that are disposed of after these were stored

Question 44: Crisps / nuts.
In your household, how much crisps / nuts were disposed of in the past week?
A portion is a handful of crisps or nuts.
- o Approximately half a portion or less
- o Approximately one portion
- o 2 to 3 portions
- o 4 to 5 portions
- o More than 5 portions

Question 45: To which category did the (majority of) disposed of crisps / nuts belong?
Please tick the category that occurred the most. You can tick more than one box if multiple categories occurred in the same amount.
- ☐ Completely unused foods: food that is disposed of which is not used at all (e.g., bag of crisps)
- ☐ Partly used foods: food that is disposed of after it is party used (e.g., half a bag of crisps)
- ☐ Meal leftovers: meal leftovers that are disposed of after these were left on the plate, pots or pans
- ☐ Leftovers after storing: meal leftovers that are disposed of after these were stored

Question 46: Non-alcoholic beverages (milk, juice, soda; excluded: water, tea, coffee, diluted syrup).
In your household, how much non-alcoholic beverages (milk, juice, soda; excluded: water, tea, coffee, diluted syrup) was disposed of in the past week?
- o Less than half a glass
- o A half to one and a half glass
- o Multiple glasses (approximately half a litre)
- o Approximately one litre
- o More than one litre

Question 47: Alcoholic beverages.
To which category did the (majority of) non-alcoholic beverages belong?
Please tick the category that occurred the most. You can tick more than one box if multiple categories occurred in the same amount.

- ☐ Completely unused foods: drinks that are disposed of which are not used at all (e.g., a milk package)
- ☐ Partly used foods: drinks that is disposed of after it is party used (e.g., half a milk package)
- ☐ Meal leftovers: beverage that is left in the glass
- ☐ Leftovers after storing: meal leftovers that are disposed of after these were stored

Question 48: Alcoholic beverages.
In your household, how many alcoholic beverages were disposed of in the past week?

- o Less than half a beer glass
- o Half to one and a half beer glass
- o Multiple beer glasses (approximately half a litre)
- o Approximately one litre
- o More than one litre

Question 49: To which category did the (majority of) alcoholic beverages belong?
Please tick the category that occurred the most. You can tick more than one box if multiple categories occurred in the same amount.

- ☐ Completely unused foods: drinks that are disposed of which are not used at all (e.g., a bottle of wine)
- ☐ Partly used foods: drinks that are disposed of after it is party used (e.g., half a bottle of wine)
- ☐ Meal leftovers: beverage that is left in the glass
- ☐ Leftovers after storing: beverage leftovers that are disposed of after these were stored

Appendix B: Household Food Waste Questionnaire in Dutch

Introduction

Vorige week heeft u een email ontvangen met het verzoek om op te letten welke voedsel en drink producten u heeft weggegooid. Deze vragenlijst zal gaan over deze producten.
Ter herinnering:

Deze vragenlijst **zal** gaan over:

- Alle eetbare voedsel en drink producten die u in de (online) (super)markt heeft gekocht of thuis heeft geteeld die zijn weggegooid.
- Inclusief, producten die bedorven of over de datum waren.
- Het maakt hierbij niet uit of u het voedsel heeft weggegooid in de afvalbak, gft-bak, op de composthoop heeft gegooid, aan (huis)dieren (huisdieren, vogels, et cetera) heeft gegeven, of op een andere manier: het moet allemaal worden meegenomen.

Deze vragenlijst zal **niet** gaan over:

- Botten, schillen, zaden of stronken.
- Voedsel of drink producten die zijn weggegooid bij het eten in een restaurant of kantine.

Questionnaire

Question 1: Klikt u nu hieronder aan welke producten er in uw huishouden de afgelopen week zijn weggegooid. In het geval van maaltijden, klik dan op verschillende hoofdingrediënten.

- ☐ Verse groenten en salade
- ☐ Niet-verse groenten (pot / blik / diepvries)
- ☐ Vers fruit
- ☐ Niet-vers fruit (pot / blik / gedroogd / diepvries)
- ☐ Aardappelen
- ☐ Aardappelproducten (frietjes, chips, voorgekookte aardappelen of krieltjes, et cetera.)
- ☐ Pasta
- ☐ Rijst en resterende graanproducten (inclusief wraps, couscous, et cetera)
- ☐ Bonen, linzen, kikkererwten et cetera
- ☐ Vlees
- ☐ Vleesvervangers
- ☐ Vis
- ☐ Broodbeleg (vleeswaren, plakjes kaas, zoet beleg, et cetera)
- ☐ Brood
- ☐ Ontbijtgranen (muesli, cruesli, brinta, et cetera)
- ☐ Yoghurt, vla, et cetera
- ☐ Kaas (blokjes kaas, Franse kaasjes, geraspte kaas. Hier valt NIET onder: broodbeleg uitgezonderd)
- ☐ Eieren
- ☐ Soepen en curry
- ☐ Sauzen (ketchup, mayonnaise, cocktailsaus, et cetera)
- ☐ Snoep / koekjes / tussendoortjes / chocolade repen
- ☐ Chips / nootjes
- ☐ Niet-alcoholische dranken (melk*, sappen, frisdrank. Hier valt NIET onder water/thee/koffie/limonade)
- ☐ Alcoholische dranken
- ☐ Ik heb geen producten weggegooid

* *NOTE*: Due to progressive insights, creating a separate category for “Milk” can be considered, as this is a frequently disposed of food product. However, please keep in mind that this will change the set-up slightly, when comparing the results with the earlier applications of this survey.

Introduction to the next part of the questionnaire

Food waste states
Wij splitsen voedselverspilling in verschillende categorieën, die hieronder staan uitgelegd. Lees dit alstublieft zorgvuldig door, in de volgende vragen zullen deze categorieën gebruikt worden.

1) Geheel ongebruikt voedsel: Voedsel dat nog helemaal niet is gebruikt. Bijvoorbeeld ongeopende verpakkingen, inclusief ongeopende delen van groot verpakkingen, beschimmelde appels, uitgedroogde prei, een heel brood.
2) Deels gebruikt voedsel: Voedsel dat weggegooid wordt nadat het deels gebruikt is. Bijvoorbeeld broodkapjes, halve verpakking vleeswaren, een halve ui of een half pak melk.
3) Maaltijdresten: Maaltijdresten die zijn weggegooid nadat deze waren overgebleven op het bord of in de pannen. Bijvoorbeeld restjes aardappelpuree of rijst op het bord of in de pan, of brood dat niet is gegeten tijdens de lunch.
4) Restjes na bewaring: Restjes of kliekjes die weggegooid worden, nadat ze bewaard zijn geweest in de koelkast of vriezer. Bijvoorbeeld, bevroren restjes pasta van vorige week.

U zult meerdere vragen krijgen over de verschillende typen eten en drinken die u hebt weggegooid in de afgelopen week. Eerst zult u worden gevraagd hoeveel u van een bepaald product hebt weggegooid in de afgelopen week. Vervolgens vragen we u in welke categorie (ongebruikt, deels gebruikt, maaltijdresten, restjes na bewaring) het meeste van het eten uit die categorie dat was weggegooid behoorde. Let alstublieft goed op over welk voedsel het gaat!

NOTE: The next questions are only shown if respondents indicated waste in the respective category in Question 1

Question 2: Verse groentes
Hoeveel verse groenten en salades zijn er in de afgelopen week weggegooid in uw huishouden?
Eén opscheplepel is 50 gram. Dit staat gelijk aan bijvoorbeeld een halve prei of vier champignons.
- o Minder dan een opscheplepel
- o 1 tot 2 opscheplepels
- o 2 tot 4 opscheplepels
- o 4 tot 6 opscheplepels
- o Meer dan 6 opscheplepels

Question 3: Verse groentes
In welke categorie viel (het merendeel van) de verse groenten en salades die zijn weggegooid?
Kruis de categorie aan die het meest voorkwam. Als er meerdere categorieën evenveel voorkwamen, kunt u meerdere antwoorden aankruisen.
- ☐ Geheel ongebruikt voedsel: Voedsel dat nog helemaal niet is gebruikt (bijvoorbeeld een hele prei)
- ☐ Deels gebruikt voedsel: Voedsel dat weggegooid wordt als het deels gebruikt is (bijvoorbeeld halve ui)
- ☐ Maaltijdresten: Maaltijdresten die op het bord of in de pan blijven liggen na het eten
- ☐ Restjes na bewaring: Restjes / kliekjes die weggegooid worden, nadat ze bewaard zijn geweest

Question 4: Niet verse groentes
Hoeveel niet-verse groenten (pot / blik / diepvries) zijn er in de afgelopen week weggegooid in uw huishouden?
Een opscheplepel is 50 gram. Dit staat gelijk aan bijvoorbeeld een halve prei of vier champignons.
- o Minder dan een opscheplepel
- o 1 tot 2 opscheplepels
- o 2 tot 4 opscheplepels
- o 4 tot 6 opscheplepels
- o Meer dan 6 opscheplepels

Question 5: Niet verse groentes
In welke categorie viel (het merendeel van) de niet-verse groenten die zijn weggegooid?
Kruis de categorie aan die het meest voorkwam. Als er meerdere categorieën evenveel voorkwamen, kunt u meerdere antwoorden aankruisen.
- ☐ Geheel ongebruikt voedsel: Voedsel dat nog helemaal niet is gebruikt (bijvoorbeeld ongeopend pak diepvriesspinazie)
- ☐ Deels gebruikt voedsel: Voedsel dat weggegooid wordt als het deels gebruikt is (bijvoorbeeld half gebruikt pak diepvriesspinazie)
- ☐ Maaltijdresten: Maaltijdresten die op het bord of in de pan blijven liggen na het eten
- ☐ Restjes na bewaring: Restjes / kliekjes die weggegooid worden, nadat ze bewaard zijn geweest

Question 6: Vers fruit
Hoeveel vers fruit is in de afgelopen week weggegooid in uw huishouden? Een appel of banaan is bijvoorbeeld een stuk fruit. Van heel klein fruit, zoals aardbeien en druiven, telt een schaaltje als '1 stuk'.
- o Ongeveer een kwart stuk fruit of minder
- o Ongeveer een half stuk fruit
- o Ongeveer 1 stuk fruit
- o 2 tot 4 stuks fruit
- o Meer dan 4 stuks fruit

Question 7: Vers fruit
In welke categorie viel (het merendeel van) het verse fruit dat is weggegooid?
Kruis de categorie aan die het meest voorkwam. Als er meerdere categorieën evenveel voorkwamen, kunt u meerdere antwoorden aankruisen.
- ☐ Geheel ongebruikt voedsel: Voedsel dat nog helemaal niet is gebruikt (bijvoorbeeld hele appel)
- ☐ Deels gebruikt voedsel: Voedsel dat weggegooid wordt als het deels gebruikt is (bijvoorbeeld halve appel die niet in gerecht wordt gebruikt)
- ☐ Etensresten: etensresten die overblijven na het eten (bijvoorbeeld half opgegeten appel of een fruitsalade)
- ☐ Restjes na bewaring: Restjes / kliekjes die weggegooid worden, nadat ze bewaard zijn geweest (e.g. een fruitsalade nadat deze was bewaard)

Question 8: Niet vers fruit
Hoeveel niet-vers fruit (pot / blik / gedroogd / diepvries) is in de afgelopen week weggegooid in uw huishouden? Een peer of perzik uit blik is bijvoorbeeld een stuk fruit. In het geval van heel klein fruit, of voorgesneden fruit zoals bosbessen en mandarijnpartjes, telt een schaaltje als '1 stuk'.
- o Ongeveer een kwart stuk fruit of minder
- o Ongeveer een half stuk fruit
- o Ongeveer 1 stuk fruit
- o 2 tot 4 stuks fruit
- o Meer dan 4 stuks fruit

Question 9: Niet vers fruit
In welke categorie viel (het merendeel van) het niet-verse fruit dat is weggegooid?
Kruis de categorie aan die het meest voorkwam. Als er meerdere categorieën evenveel voorkwamen, kunt u meerdere antwoorden aankruisen.
- ☐ Geheel ongebruikt voedsel: Voedsel dat nog helemaal niet is gebruikt (bijvoorbeeld ongeopend blik fruit)
- ☐ Deels gebruikt voedsel: Voedsel dat weggegooid wordt als het deels gebruikt is (bijvoorbeeld halfvol blik fruit)
- ☐ Maaltijdresten: Maaltijdresten die op het bord of in de pan blijven liggen na het eten (bijvoorbeeld een schaaltje met fruit)
- ☐ Restjes na bewaring: Restjes / kliekjes die weggegooid worden, nadat ze bewaard zijn geweest (bijvoorbeeld een fruitsalade nadat deze was bewaard)

Question 10: Aardappelen
Hoeveel aardappelen zijn in de afgelopen week weggegooid in uw huishouden? Één opscheplepel is 50 gram. Dit staat gelijk aan bijvoorbeeld een halve middelgrote aardappel.
- o Minder dan een opscheplepel
- o 1 tot 2 opscheplepels
- o 2 tot 4 opscheplepels
- o 4 tot 6 opscheplepels
- o Meer dan 6 opscheplepels

Question 11: Aardappelen
In welke categorie viel (het merendeel van) de aardappelen die zijn weggegooid?
Kruis de categorie aan die het meest voorkwam. Als er meerdere categorieën evenveel voorkwamen, kunt u meerdere antwoorden aankruisen.
- ☐ Geheel ongebruikt voedsel: Voedsel dat nog helemaal niet is gebruikt (bijvoorbeeld heel pak aardappelen)
- ☐ Deels gebruikt voedsel: Voedsel dat weggegooid wordt als het deels gebruikt is (bijvoorbeeld half pak aardappelen)
- ☐ Maaltijdresten: Maaltijdresten die op het bord of in de pan blijven liggen na het eten (bijvoorbeeld aardappelpuree)
- ☐ Restjes na bewaring: Restjes / kliekjes die weggegooid worden, nadat ze bewaard zijn geweest (bijvoorbeeld aardappelpuree nadat het is bewaard)

Question 12: Aardappelproducten
Hoeveel aardappelproducten (frietjes, voorgekookte krieltjes, et cetera) zijn in de afgelopen week weggegooid in uw huishouden?
- o Minder dan 10 frietjes / krieltjes / stuks
- o 10 tot 25 frietjes / krieltjes / stuks
- o Meer dan 25 frietjes / krieltjes / et cetera (ongeveer een half pak van 750 gram)
- o Een heel pak (750 gram) frietjes / krieltjes / et cetera
- o Meer dan een heel pak (750 gram) frietjes / krieltjes / et cetera

Question 13: Aardappelproducten
In welke categorie viel (het merendeel van) de aardappelproducten die zijn weggegooid?
Kruis de categorie aan die het meest voorkwam. Als er meerdere categorieën evenveel voorkwamen, kunt u meerdere antwoorden aankruisen.
- ☐ Geheel ongebruikt voedsel: Voedsel dat nog helemaal niet is gebruikt (bijvoorbeeld heel pak frietjes)
- ☐ Deels gebruikt voedsel: Voedsel dat weggegooid wordt als het deels gebruikt is (bijvoorbeeld half pak frietjes)
- ☐ Maaltijdresten: Maaltijdresten die op het bord of in de pan blijven liggen na het eten
- ☐ Restjes na bewaring: Restjes / kliekjes die weggegooid worden, nadat ze bewaard zijn geweest

Question 14: Pasta
Hoeveel pasta is in de afgelopen week weggegooid in uw huishouden?
Eén opscheplepel is 50 gram.
- o Minder dan een opscheplepel
- o 1 tot 2 opscheplepels
- o 2 tot 4 opscheplepels
- o 4 tot 6 opscheplepels
- o Meer dan 6 opscheplepels

Question 15: Pasta
In welke categorie viel (het merendeel van) de pasta die is weggegooid?
Kruis de categorie aan die het meest voorkwam. Als er meerdere categorieën evenveel voorkwamen, kunt u meerdere antwoorden aankruisen.
- ☐ Geheel ongebruikt voedsel: Voedsel dat nog helemaal niet is gebruikt (bijvoorbeeld heel pak pasta)
- ☐ Deels gebruikt voedsel: Voedsel dat weggegooid wordt als het deels gebruikt is (bijvoorbeeld half pak pasta)
- ☐ Maaltijdresten: Maaltijdresten die op het bord of in de pan blijven liggen na het eten (bijvoorbeeld gekookte pasta)
- ☐ Restjes na bewaring: Restjes / kliekjes die weggegooid worden, nadat ze bewaard zijn geweest (bijvoorbeeld gekookte pasta nadat het bewaard was)

Question 16: Rijst
Hoeveel rijst en andere graanproducten (inclusief wraps, couscous, et cetera) zijn er in de afgelopen week weggegooid in uw huishouden?
Eén opscheplepel is 50 gram.
- o Minder dan een opscheplepel
- o 1 tot 2 opscheplepels
- o 2 tot 4 opscheplepels
- o 4 tot 6 opscheplepels
- o Meer dan 6 opscheplepels

Question 17: Rijst
In welke categorie viel (het merendeel van) de rijst andere graanproducten die is weggegooid?
Kruis de categorie aan die het meest voorkwam. Als er meerdere categorieën evenveel voorkwamen, kunt u meerdere antwoorden aankruisen.
- ☐ Geheel ongebruikt voedsel: Voedsel dat nog helemaal niet is gebruikt (bijvoorbeeld heel pak rijst)
- ☐ Deels gebruikt voedsel: Voedsel dat weggegooid wordt als het deels gebruikt is (bijvoorbeeld half pak rijst)
- ☐ Maaltijdresten: Maaltijdresten die op het bord of in de pan blijven liggen na het eten (bijvoorbeeld gekookte rijst)
- ☐ Restjes na bewaring: Restjes / kliekjes die weggegooid worden, nadat ze bewaard zijn geweest (bijvoorbeeld gekookte rijst nadat deze bewaard was)

Question 18: Bonen
Hoeveel bonen, linzen, kikkererwten et cetera zijn er in de afgelopen week weggegooid in uw huishouden?
Eén opscheplepel is 50 gram.

- Minder dan een opscheplepel
- 1 tot 2 opscheplepels
- 2 tot 4 opscheplepels
- 4 tot 6 opscheplepels
- Meer dan 6 opscheplepels

Question 19: Bonen
In welke categorie viel (het merendeel van) de bonen, linzen, kikkererwten et cetera die is weggegooid?
Kruis de categorie aan die het meest voorkwam. Als er meerdere categorieën evenveel voorkwamen, kunt u meerdere antwoorden aankruisen.

- Geheel ongebruikt voedsel: Voedsel dat nog helemaal niet is gebruikt (bijvoorbeeld een ongeopend blik bonen)
- Deels gebruikt voedsel: Voedsel dat weggegooid wordt als het deels gebruikt is (bijvoorbeeld half vol blik bonen)
- Maaltijdresten: Maaltijdresten die op het bord of in de pan blijven liggen na het eten
- Restjes na bewaring: Restjes / kliekjes die weggegooid worden, nadat ze bewaard zijn geweest

Question 20: Vlees
Hoeveel vlees is er in de afgelopen week weggegooid in uw huishouden?
Een portie is bijvoorbeeld 1 kipfilet, 1 biefstuk, et cetera. Question: Hoeveelheid van stukjes vlees, bijvoorbeeld gehakt, graag zo goed als het kan vertalen naar Question: Hoeveelheid hele stukken vlees. (Een pak gehakt is ongeveer twee porties).

- Ongeveer een halve portie of minder
- Ongeveer een hele portie
- 2 tot 3 porties
- 4 tot 5 porties
- Meer dan 5 porties

Question 21: Vlees
In welke categorie viel (het merendeel van) het vlees dat is weggegooid?
Kruis de categorie aan die het meest voorkwam. Als er meerdere categorieën evenveel voorkwamen, kunt u meerdere antwoorden aankruisen.

- Geheel ongebruikt voedsel: Voedsel dat nog helemaal niet is gebruikt (bijvoorbeeld pak worstjes)
- Deels gebruikt voedsel: Voedsel dat weggegooid wordt als het deels gebruikt is (bijvoorbeeld half pak worstjes)
- Maaltijdresten: Maaltijdresten die op het bord of in de pan blijven liggen na het eten
- Restjes na bewaring: Restjes / kliekjes die weggegooid worden, nadat ze bewaard zijn geweest

Question 22: Vleesvervangers
Hoeveel vleesvervangers zijn er in de afgelopen week weggegooid in uw huishouden? Een portie is bijvoorbeeld een vegetarische burger. Probeer in het geval van stukjes, bijvoorbeeld vegetarisch gehakt, deze zo goed mogelijk te vertalen naar Question: Hoeveelheid hele stukken vleesvervanger. Zo staat een pak vegetarisch gehakt gelijk aan twee porties.

- Ongeveer een halve portie of minder
- Ongeveer een hele portie
- 2 tot 3 porties
- 4 tot 5 porties
- Meer dan 5 porties

Question 23: Vleesvervangers
In welke categorie viel (het merendeel van) de vleesvervangers die zijn weggegooid? Kruis de categorie aan die het meest voorkwam. Als er meerdere categorieën evenveel voorkwamen, kunt u meerdere antwoorden aankruisen.

- Geheel ongebruikt voedsel: Voedsel dat nog helemaal niet is gebruikt (bijvoorbeeld pak vegetarische burgers)
- Deels gebruikt voedsel: Voedsel dat weggegooid wordt als het deels gebruikt is (bijvoorbeeld half pak vegetarische burgers)
- Maaltijdresten: Maaltijdresten die op het bord of in de pan blijven liggen na het eten
- Restjes na bewaring: Restjes / kliekjes die weggegooid worden, nadat ze bewaard zijn geweest

Question 24: Vis
Hoeveel vis is in de afgelopen week weggegooid in uw huishouden? Een portie is bijvoorbeeld 1 visfilet, 1 stuk zalm, et cetera.
- Ongeveer een halve portie of minder
- Ongeveer een hele portie
- 2 tot 3 porties
- 4 tot 5 porties
- Meer dan 5 porties

Question 25: Vis
In welke categorie viel (het merendeel van) de vis die is weggegooid? Kruis de categorie aan die het meest voorkwam. Als er meerdere categorieën evenveel voorkwamen, kunt u meerdere antwoorden aankruisen.
- Geheel ongebruikt voedsel: Voedsel dat nog helemaal niet is gebruikt (bijvoorbeeld heel pakje vis)
- Deels gebruikt voedsel: Voedsel dat weggegooid wordt als het deels gebruikt is (bijvoorbeeld half pakje vis)
- Maaltijdresten: Maaltijdresten die op het bord of in de pan blijven liggen na het eten
- Restjes na bewaring: Restjes / kliekjes die weggegooid worden, nadat ze bewaard zijn geweest

Question 26: Broodbeleg
Hoeveel broodbeleg (vleeswaren, zoet beleg, plakjes kaas, et cetera) is in de afgelopen week weggegooid in uw huishouden? Een portie is de Question: Hoeveelheid broodbeleg die op 1 boterham gebruikt wordt.
- Ongeveer een halve portie of minder
- Ongeveer een hele portie
- 2 tot 3 porties
- 4 tot 5 porties
- Meer dan 5 porties

Question 27: Broodbeleg
In welke categorie viel (het merendeel van) het broodbeleg dat is weggegooid? Kruis de categorie aan die het meest voorkwam. Als er meerdere categorieën evenveel voorkwamen, kunt u meerdere antwoorden aankruisen.
- Geheel ongebruikt voedsel: Voedsel dat nog helemaal niet is gebruikt (bijvoorbeeld heel pakje vleeswaren)
- Deels gebruikt voedsel: Voedsel dat weggegooid wordt als het deels gebruikt is (bijvoorbeeld half pakje vleeswaren)
- Maaltijdresten: Maaltijdresten die op het bord of in de pan blijven liggen na het eten (bijvoorbeeld boterhambeleg dat na de lunch wordt weggegooid (eventueel brood))
- Restjes na bewaring: Restjes / kliekjes die weggegooid worden, nadat ze bewaard zijn geweest (bijvoorbeeld broodbeleg (eventueel met brood) nadat deze was bewaard)

Question 28: Brood
Hoeveel brood is in de afgelopen week weggegooid in uw huishouden? Een bolletje / pistolet / krentenbol / et cetera kunt u gelijkstellen aan een sneetje brood.
- Minder dan een sneetje brood
- Eén of enkele sneetjes brood
- Ongeveer een half brood
- Ongeveer een heel brood
- Meer dan een heel brood

Question 29: Brood
In welke categorie viel (het merendeel van) het brood dat is weggegooid? Kruis de categorie aan die het meest voorkwam. Als er meerdere categorieën evenveel voorkwamen, kunt u meerdere antwoorden aankruisen.
- Geheel ongebruikt voedsel: Voedsel dat nog helemaal niet is gebruikt (bijvoorbeeld heel brood)
- Deels gebruikt voedsel: Voedsel dat weggegooid wordt als het deels gebruikt is (bijvoorbeeld paar sneden brood)
- Maaltijdresten: Maaltijdresten die op het bord blijven liggen na het eten (bijvoorbeeld broodkorstjes op het bord)
- Restjes na bewaring: Restjes / kliekjes die weggegooid worden, nadat ze bewaard zijn geweest (bijvoorbeeld een belegde boterham nadat deze was bewaard)

Question 30: Ontbijtgranen
Hoeveel ontbijtgranen (muesli, cruesli, brinta, et cetera) is in de afgelopen week weggegooid in uw huishouden? Een portie is de Question: Hoeveelheid van een bakje ontbijtgranen wat als ontbijt wordt gegeten.
- Minder dan een halve portie
- Een halve tot anderhalve portie
- Meerdere porties (ongeveer een half pak)
- Ongeveer een heel pak
- Meerdere pakken

Question 31: Ontbijtgranen
In welke categorie viel (het merendeel van) de ontbijtgranen die zijn weggegooid? Kruis de categorie aan die het meest voorkwam. Als er meerdere categorieën evenveel voorkwamen, kunt u meerdere antwoorden aankruisen.
- Geheel ongebruikt voedsel: Voedsel dat nog helemaal niet is gebruikt (bijvoorbeeld heel pak muesli)
- Deels gebruikt voedsel: Voedsel dat weggegooid wordt als het deels gebruikt is (bijvoorbeeld half pak muesli)
- Maaltijdresten: Maaltijdresten die op het bord / in de kom blijven liggen na het eten
- Restjes na bewaring: Restjes / kliekjes die weggegooid worden, nadat ze bewaard zijn geweest

Question 32: Yoghurt
Hoeveel yoghurt, vla, et cetera is in de afgelopen week weggegooid in uw huishouden? Een portie is een dessertschaaltje met yoghurt / vla / et cetera.
- Minder dan een halve portie
- Een halve tot anderhalve portie
- Meerdere porties (ongeveer een half literpak)
- Ongeveer een heel literpak
- Meerdere literpakken

Question 33: Yoghurt
In welke categorie viel (het merendeel van) de yoghurt, vla, et cetera. die zijn weggegooid? Kruis de categorie aan die het meest voorkwam. Als er meerdere categorieën evenveel voorkwamen, kunt u meerdere antwoorden aankruisen.

- Geheel ongebruikt voedsel: Voedsel dat nog helemaal niet is gebruikt (bijvoorbeeld heel pak vla)
- Deels gebruikt voedsel: Voedsel dat weggegooid wordt als het deels gebruikt is (bijvoorbeeld half pak vla)
- Maaltijdresten: Maaltijdresten die op het bord blijven liggen na het eten
- Restjes na bewaring: Restjes / kliekjes die weggegooid worden, nadat ze bewaard zijn geweest

Question 34: Kaas
Hoeveel kaas (broodbeleg uitgezonderd) is in de afgelopen week weggegooid in uw huishouden? Een handje vol strooikaas kunt u gelijkstellen aan 1 blokje kaas.
- Minder dan een blokje kaas
- Ongeveer 1 blokje kaas
- 1 tot 3 blokjes kaas
- 4 tot 5 blokjes kaas
- Meer dan 5 blokjes kaas

NOTE with question 34: Due to progressive insights, increasing the measure options can be considered, as 5 cheese dices is low compared to the potential maximum gram of waste of the other categories. However, please keep in mind that this will change the set-up slightly, when comparing the results with the earlier applications of this survey.

Question 35: Kaas
In welke categorie viel (het merendeel van) de kaas die is weggegooid? Kruis de categorie aan die het meest voorkwam. Als er meerdere categorieën evenveel voorkwamen, kunt u meerdere antwoorden aankruisen.
- Geheel ongebruikt voedsel: Voedsel dat nog helemaal niet is gebruikt (bijvoorbeeld hele brie)
- Deels gebruikt voedsel: Voedsel dat weggegooid wordt als het deels gebruikt is (bijvoorbeeld half stuk brie)
- Maaltijdresten: Maaltijdresten die op het bord blijven liggen na het eten
- Restjes na bewaring: Restjes / kliekjes die weggegooid worden, nadat ze bewaard zijn geweest

Question 36: Eieren
Hoeveel eieren zijn in de afgelopen week weggegooid in uw huishouden?
- Minder dan 1 ei
- 1 ei
- 2 tot 3 eieren
- 4 tot 5 eieren
- Meer dan 5 eieren

Question 37: Eieren
In welke categorie viel (het merendeel van) de eieren die zijn weggegooid? Kruis de categorie aan die het meest voorkwam. Als er meerdere categorieën evenveel voorkwamen, kunt u meerdere antwoorden aankruisen.
- Geheel ongebruikt voedsel: Voedsel dat nog helemaal niet is gebruikt (bijvoorbeeld hele eieren)
- Deels gebruikt voedsel: Voedsel dat weggegooid wordt als het deels gebruikt is (bijvoorbeeld eiwit)
- Maaltijdresten: Maaltijdresten die op het bord of in de pan blijven liggen na het eten
- Restjes na bewaring: Restjes / kliekjes die weggegooid worden, nadat ze bewaard zijn geweest (bijvoorbeeld een gekookt ei nadat deze was bewaard)

Question 38: Soep
Hoeveel soep en curry is in de afgelopen week weggegooid in uw huishouden?
- Minder dan een halve soeplepel
- Een halve tot anderhalve soeplepel
- Meerdere soeplepels (ongeveer een halve liter)
- Ongeveer een hele liter
- Meer dan een liter

Question 39: Soep
In welke categorie viel (het merendeel van) de soep/curry die is weggegooid? Kruis de categorie aan die het meest voorkwam. Als er meerdere categorieën evenveel voorkwamen, kunt u meerdere antwoorden aankruisen.
- Geheel ongebruikt voedsel: Voedsel dat nog helemaal niet is gebruikt (bijvoorbeeld heel pak soep)
- Deels gebruikt voedsel: Voedsel dat weggegooid wordt als het deels gebruikt is (bijvoorbeeld half pak soep)
- Maaltijdresten: Maaltijdresten die op het bord of in de pan blijven liggen na het eten
- Restjes na bewaring: Restjes / kliekjes die weggegooid worden, nadat ze bewaard zijn geweest

Question 40: Saus
Hoeveel saus (ketchup, mayonaise, cocktailsaus, et cetera) is in de afgelopen week weggegooid in uw huishouden? Één eetlepel is 20 gram.
- Minder dan een eetlepel
- 1 tot 3 eetlepels
- Meerdere eetlepels (ongeveer een halve pot / fles)
- Ongeveer een hele pot / fles
- Meer dan een hele pot / fles

Question 41: Saus
In welke categorie viel (het merendeel van) de saus die is weggegooid? Kruis de categorie aan die het meest voorkwam. Als er meerdere categorieën evenveel voorkwamen, kunt u meerdere antwoorden aankruisen.
- Geheel ongebruikt voedsel: Voedsel dat nog helemaal niet is gebruikt (bijvoorbeeld heel potje saus)
- Deels gebruikt voedsel: Voedsel dat weggegooid wordt als het deels gebruikt is (bijvoorbeeld half potje saus)
- Maaltijdresten: Maaltijdresten die op het bord of in de pan blijven liggen na het eten
- Restjes na bewaring: Restjes / kliekjes die weggegooid worden, nadat ze bewaard zijn geweest

Question 42: Tussendoortjes
Hoeveel snoep / koekjes / tussendoortjes / chocolade repen is in de afgelopen week weggegooid in uw huishouden? Een portie is een handje kleine dropjes, een kleine chocoladereep, een koekje, et cetera.
- Ongeveer een halve portie of minder
- Ongeveer een hele portie
- 2 tot 3 porties
- 4 tot 5 porties
- Meer dan 5 porties

Question 43: Tussendoortjes
In welke categorie viel (het merendeel van) de snoep / koekjes / tussendoortjes / chocolade repen die zijn weggegooid? Kruis de categorie aan die het meest voorkwam. Als er meerdere categorieën evenveel voorkwamen, kunt u meerdere antwoorden aankruisen.
- Geheel ongebruikt voedsel: Voedsel dat nog helemaal niet is gebruikt (bijvoorbeeld heel pak koekjes)
- Deels gebruikt voedsel: Voedsel dat weggegooid wordt als het deels gebruikt is (bijvoorbeeld half pak koekjes)
- Etensresten: Resten die zijn overgebleven bij het eten
- Restjes na bewaring: Restjes / kliekjes die weggegooid worden, nadat ze bewaard zijn geweest

Question 44: Chips
Hoeveel chips / nootjes is in de afgelopen week weggegooid in uw huishouden?
Een portie is een handvol chips of een handvol nootjes.
- Ongeveer een halve portie of minder
- Ongeveer een hele portie
- 2 tot 3 porties
- 4 tot 5 porties
- Meer dan 5 porties

Question 45: Chips
In welke categorie viel (het merendeel van) de chips / nootjes die zijn weggegooid? Kruis de categorie aan die het meest voorkwam. Als er meerdere categorieën evenveel voorkwamen, kunt u meerdere antwoorden aankruisen.
- Geheel ongebruikt voedsel: Voedsel dat nog helemaal niet is gebruikt (bijvoorbeeld hele zak chips)
- Deels gebruikt voedsel: Voedsel dat weggegooid wordt als het deels gebruikt is (bijvoorbeeld halve zak chips)
- Etensresten: Resten die zijn overgebleven bij het eten
- Restjes na bewaring: Restjes / kliekjes die weggegooid worden, nadat ze bewaard zijn geweest

Question 46: Niet-alcoholische drank
Hoeveel niet-alcoholische drank (melk, sappen, frisdrank, hier valt NIET onder water/thee/koffie/siroop) is in de afgelopen week weggegooid in uw huishouden?
- Minder dan een half glas
- Een half tot anderhalf glas
- Meerdere glazen (ongeveer een halve liter)
- Ongeveer een hele liter
- Meer dan een hele liter

Question 47: Niet-alcoholische drank
In welke categorie viel (het merendeel van) de niet-alcoholische drank die is weggegooid? Kruis de categorie aan die het meest voorkwam. Als er meerdere categorieën evenveel voorkwamen, kunt u meerdere antwoorden aankruisen.
- Geheel ongebruikt voedsel: Drank die nog helemaal niet is gebruikt (bijvoorbeeld heel pak melk)
- Deels gebruikt voedsel: Drank die weggegooid wordt als het deels gebruikt is (bijvoorbeeld half pak melk)
- Maaltijdresten: Drank die in het glas of beker is achtergebleven
- Restjes na bewaring: Restjes / kliekjes die weggegooid worden, nadat ze bewaard zijn geweest

Question 48: Alcoholische drank
Hoeveel alcoholische drank is in de afgelopen week weggegooid in uw huishouden?
- Minder dan een half bierglas
- Een half tot anderhalf bierglas
- Meerdere bierglazen (ongeveer een halve liter)
- Ongeveer een hele liter
- Meer dan een hele liter

Question 49: Alcoholische drank
In welke categorie viel (het merendeel van) de alcoholische drank die is weggegooid? Kruis de categorie aan die het meest voorkwam. Als er meerdere categorieën evenveel voorkwamen, kunt u meerdere antwoorden aankruisen.
- Geheel ongebruikt voedsel: Drank die nog helemaal niet is gebruikt (bijvoorbeeld hele fles wijn)
- Deels gebruikt voedsel: Drank die weggegooid wordt als het deels gebruikt is (bijvoorbeeld halve fles wijn)
- Maaltijdresten: Drank die in het glas of beker is achtergebleven
- Restjes na bewaring: Restjes / kliekjes die weggegooid worden, nadat ze bewaard zijn geweest

Appendix C: Household Food Waste Questionnaire in German

Introduction

Letzte Woche erhielten Sie eine E-Mail, um die Aufmerksamkeit auf das Essen und Trinken Produkt, das Sie weggeworfen haben, zu widmen. Dieser Fragebogen wird über diese Produkte sein. Als eine Erinnerung:

Dieser Fragebogen bezieht sich auf:

- Lebensmittel- und Getränkeprodukte, die Sie im (Online-) (Super-) Markt gekauft haben, oder selbst angebaut wurden aber schließlich entsorgt wurden.
- Dazu gehören auch Produkte, die Sie weggeworfen haben, weil sie verdorben waren oder das Mindesthaltbarkeitsdatum überschritten wurde.
- Es spielt keine Rolle, ob die Produkte im allgemeinen Hausmüll, im Container für Essenreste oder auf dem Kompost entsorgt oder an Tiere (Haustiere, Vögel, etc.) verfüttert werden, alle Arten der Entsorgung werden berücksichtigt.

Es geht nicht über:

- Knochen, Schalen, Samen und Strünke nicht.
- Lebensmittel und Getränke, die beim Essen in einem Restaurant oder einer Kantine entsorgt werden, nicht berücksichtigt.

Questionnaire

Bitte kreuzen Sie die Produkte an, die in der letzten Woche in Ihrem Haushalt entsorgt wurden. Im Falle von kompletten Mahlzeiten, benennen Sie bitte getrennt die Hauptzutaten.

☐ Frisches Gemüse und Salate
☐ Nicht- frisches Gemüse (Glas / Dose/ Tiefgefrorenes)
☐ Frisches Obst
☐ Nicht- frisches Obst (Glas / Dose / Getrocknet / Tiefgefrorenes)
☐ Kartoffeln
☐ Kartoffelprodukte (Pommes, Chips, Baby- oder vorgekochte Kartoffeln, etc.)
☐ Nudeln
☐ Reis und anderes Getreide (einschließlich Wraps, Couscous, etc.)
☐ Bohnen, Linsen, Kichererbsen etc.
☐ Fleisch (bitte kreuzen Sie für kaltes, geschnittenes Fleisch "Brotbelag" an)
☐ Fleischersatzprodukte
☐ Fisch
☐ Brotbelag (Wurst und kaltes, geschnittenes Fleisch, Käsescheiben, süße Brotaufstriche, etc.)
☐ Brot
☐ Müsli (Granola, Haferflocken, etc.)
☐ Joghurt, Pudding, etc.
☐ Käse (Käsewürfel, Käse am Stück, Streukäse, etc., nicht: Käse als Brotbelag)
☐ Eier
☐ Suppe / Eintöpfe
☐ Soßen (Ketchup, Mayonnaise, Cocktailsoße, etc.)
☐ Süßigkeiten / Kekse / Müsliriegel / Schokoriegel
☐ Cracker / Nüsse
☐ Nicht- alkoholische Getränke (Milch*, Saft, Softdrinks, nicht: Wasser, Tee, Kaffee, Sirup)
☐ Alkoholische Getränke
☐ Ich habe keine Lebensmittel- oder Getränkeprodukte entsorgt.

* *NOTE*: Due to progressive insights, creating a separate category for "Milk" can be considered, as this is a frequently disposed of food product. However, please keep in mind that this will change the set-up slightly, when comparing the results with the earlier applications of this survey.

Introduction to the next part of the questionnaire

Wir teilen Lebensmittelabfälle in mehrere Kategorien auf, die nachfolgend erläutert werden. Bitte lesen Sie diese sorgfältig, da diese Kategorien in den nächsten Fragen verwendet werden.

1) <u>Komplett unverbrauchte Lebensmittel</u>: Lebensmittel, die entsorgt werden, die überhaupt nicht verwendet wurden. Zum Beispiel ungeöffnete Packungen, inkl. ungeöffnete Bestandteile von Sammelpackungen, verfaulte Äpfel, vertrockneter Lauch, kompletter Brotlaib
2) <u>Teilweise verbrauchte Lebensmittel</u>: Lebensmittel, die entsorgt werden, nachdem sie teilweise verbraucht wurden. Zum Beispiel ein paar Brotscheiben, eine halbe Packung Wurstscheiben, eine halbe Zwiebel, eine halbe Packung Milch.
3) <u>Speisereste</u>: Lebensmittelreste, die entsorgt werden, nachdem sie auf dem Teller, im Topf oder in der Pfanne übriggelassen wurden. Zum Beispiel Kartoffelpüree oder Reis, dass auf dem Teller oder im Topf übriggelassen wurde, belegtes Brötchen, das nicht gegessen wurde.
4) <u>Speisereste nach Aufbewahrung</u>: Speisereste, die entsorgt werden, nachdem sie im Kühlschrank oder in der Tiefkühltruhe aufbewahrt wurden, um später verbraucht zu werden. Zum Beispiel eine Portion gefrorene Nudeln von letzter Woche.

Sie erhalten mehrere Fragen über verschiedene Arten von Lebensmittel- und Getränkeprodukte, die Sie in der vergangenen Woche entsorgt haben. Zuerst fragen wir, wie viel von einem bestimmten Produkt in Ihrem Haushalt in der vergangenen Woche entsorgt wurde. Als nächstes fragen wir, welcher Kategorie (unverbraucht, teilweise verbraucht, Speisereste, Speisereste nach Aufbewahrung) die Mehrheit der entsorgten Lebensmittelprodukte angehörten, als sie entsorgt wurden. Bitte beachten Sie, auf welches Lebensmittelprodukt sich die Frage bezieht!

NOTE: The next questions are only shown if respondents indicated waste in the respective category in Question 1

Question 2: Frischen Gemüse
In Ihrem Haushalt, wie viel frisches Gemüse wurde in der vergangenen Woche entsorgt?
Ein Servierlöffel entspricht 50 g. Ein Anhaltspunkt: das ist ungefähr eine halbe Lauchstange oder 4 Pilze.

- o Weniger als ein Servierlöffel
- o 1 bis 2 Servierlöffel
- o 2 bis 4 Servierlöffel
- o 4 bis 6 Servierlöffel
- o Mehr als 6 Servierlöffel

Question 3: Frischen Gemüse
Zu welcher Kategorie hat die (Mehrheit) des entsorgten frischen Gemüses und Salats gehört? *Bitte kreuzen Sie die Kategorie an, die am meisten aufgetreten ist. Sie können mehr als ein Kästchen ankreuzen, wenn mehrere Kategorien in der gleichen Menge aufgetreten sind.*

- □ Komplett unverbrauchte Lebensmittel: Lebensmittel, die entsorgt werden, die überhaupt nicht verwendet wurden (z. B. eine Stange Lauch).
- □ Teilweise verbrauchte Lebensmittel: Lebensmittel, die entsorgt werden, nachdem sie teilweise verbraucht wurden (z.B. eine halbe Zwiebel).
- □ Speisereste: Lebensmittelreste, die entsorgt werden, nachdem sie auf dem Teller, im Topf oder in der Pfanne übriggelassen wurden.
- □ Speisereste nach Aufbewahrung: Speisereste, die entsorgt werden, nachdem sie im Kühlschrank oder in der Tiefkühltruhe aufbewahrt wurden.

Question 4: Nicht- frisches Gemüse
In Ihrem Haushalt, wie viel nicht- frisches Gemüse (Glas / Dose / Tiefgefrorenes) wurde in der vergangenen Woche entsorgt?
Ein Servierlöffel entspricht 50 g. Ein Anhaltspunkt: das ist ungefähr eine halbe Lauchstange oder 4 Pilze.

- o Weniger als ein Servierlöffel
- o 1 bis 2 Servierlöffel
- o 2 bis 4 Servierlöffel
- o 4 bis 6 Servierlöffel
- o Mehr als 6 Servierlöffel

Question 5: Nicht- frisches Gemüse
Zu welcher Kategorie hat die (Mehrheit) des entsorgten nicht- frischen Gemüses gehört? *Bitte kreuzen Sie die Kategorie an, die am meisten aufgetreten ist. Sie können mehr als ein Kästchen ankreuzen, wenn mehrere Kategorien in der gleichen Menge aufgetreten sind.*

- ☐ Komplett unverbrauchte Lebensmittel: Lebensmittel, die entsorgt werden, die überhaupt nicht verwendet wurden (z. B. ungeöffnete tiefgekühlte Packung Spinat / ungeöffnete Dose Spinat).
- ☐ Teilweise verbrauchte Lebensmittel: Lebensmittel, die entsorgt werden, nachdem sie teilweise verbraucht wurden (z.B. halbe Packung tiefgekühlter Spinat, halbe Dose Spinat).
- ☐ Speisereste: Lebensmittelreste, die entsorgt werden, nachdem sie auf dem Teller, im Topf oder in der Pfanne übriggelassen wurden.
- ☐ Speisereste nach Aufbewahrung: Speisereste, die entsorgt werden, nachdem sie im Kühlschrank oder in der Tiefkühltruhe aufbewahrt wurden.

Question 6: Frisches Obst
In Ihrem Haushalt, wie viel frisches Obst wurde in der vergangenen Woche entsorgt?
Ein Apfel oder eine Banane ist ein Stück Obst. Im Fall von kleinen Früchten, wie Erdbeeren oder Trauben, zählt eine kleine Schale als ein Stück.

- o Ungefähr ein Viertel eines Stückes Obst oder weniger
- o Ungefähr ein halbes Stück Obst
- o Ungefähr ein Stück Obst
- o 2 bis 4 Stücke Obst
- o Mehr als 4 Stücke Obst

Question 7: Frisches Obst
Zu welcher Kategorie hat die (Mehrheit) des entsorgten frischen Obstes gehört?
Bitte kreuzen Sie die Kategorie an, die am meisten aufgetreten ist. Sie können mehr als ein Kästchen ankreuzen, wenn mehrere Kategorien in der gleichen Menge aufgetreten sind.

- ☐ Komplett unverbrauchte Lebensmittel: Lebensmittel, die entsorgt werden, die überhaupt nicht verwendet wurden (z. B. ein Apfel).
- ☐ Teilweise verbrauchte Lebensmittel: Lebensmittel, die entsorgt werden, nachdem sie teilweise verbraucht wurden (z.B. ein halber Apfel, der nicht in einem Gericht verwendet wird).
- ☐ Speisereste: Lebensmittelreste, die entsorgt werden, nachdem sie auf dem Teller, im Topf oder in der Pfanne übriggelassen wurden (z.B. halb gegessener Apfel oder Obstsalat).
- ☐ Speisereste nach Aufbewahrung: Speisereste, die entsorgt werden, nachdem sie im Kühlschrank oder in der Tiefkühltruhe aufbewahrt wurden (z.B. aufbewahrter Obstsalat).

Question 8: Nicht- frisches Obst
In Ihrem Haushalt, wie viel nicht- frisches Obst wurde in der vergangenen Woche entsorgt?
Eine Birne oder ein Pfirsich aus der Dose ist ein Stück Obst. Im Fall von kleinen Früchten, wie Blaubeeren oder Mandarinenstücken, zählt eine kleine Schale als ein Stück.

- o Ungefähr ein Viertel eines Stückes Obst oder weniger
- o Ungefähr ein halbes Stück Obst
- o Ungefähr ein Stück Obst
- o 2 bis 4 Stücke Obst
- o Mehr als 4 Stücke Obst

Question 9: Nicht- frisches Obst
Zu welcher Kategorie hat die (Mehrheit) des entsorgten nicht- frischen Obstes gehört?
Bitte kreuzen Sie die Kategorie an, die am meisten aufgetreten ist. Sie können mehr als ein Kästchen ankreuzen, wenn mehrere Kategorien in der gleichen Menge aufgetreten sind.

- ☐ Komplett unverbrauchte Lebensmittel: Lebensmittel, die entsorgt werden, die überhaupt nicht verwendet wurden (z. B. eine ungeöffnete Dose Obst).
- ☐ Teilweise verbrauchte Lebensmittel: Lebensmittel, die entsorgt werden, nachdem sie teilweise verbraucht wurden (z.B. halbe Dose Obst).
- ☐ Speisereste: Lebensmittelreste, die entsorgt werden, nachdem sie auf dem Teller, im Topf oder in der Pfanne übriggelassen wurden (z.B. Schüssel mit Obst)
- ☐ Speisereste nach Aufbewahrung: Speisereste, die entsorgt werden, nachdem sie im Kühlschrank oder in der Tiefkühltruhe aufbewahrt wurden (z.B. aufbewahrter Obstsalat).

Question 10: Kartoffeln
In Ihrem Haushalt, wie viele Kartoffeln wurden in der vergangenen Woche entsorgt?
Ein Servierlöffel entspricht 50 g. Ein Anhaltspunkt: das ist ungefähr eine halbe Lauchstange oder 4 Pilze.
- Weniger als ein Servierlöffel
- 1 bis 2 Servierlöffel
- 2 bis 4 Servierlöffel
- 4 bis 6 Servierlöffel
- Mehr als 6 Servierlöffel

Question 11: Kartoffeln
Zu welcher Kategorie hat die (Mehrheit) der entsorgten Kartoffeln gehört?
Bitte kreuzen Sie die Kategorie an, die am meisten aufgetreten ist. Sie können mehr als ein Kästchen ankreuzen, wenn mehrere Kategorien in der gleichen Menge aufgetreten sind.
- ☐ Komplett unverbrauchte Lebensmittel: Lebensmittel, die entsorgt werden, die überhaupt nicht verwendet wurden (z. B. komplette Packung Kartoffeln).
- ☐ Teilweise verbrauchte Lebensmittel: Lebensmittel, die entsorgt werden, nachdem sie teilweise verbraucht wurden (z.B. halbe Packung Kartoffeln).
- ☐ Speisereste: Lebensmittelreste, die entsorgt werden, nachdem sie auf dem Teller, im Topf oder in der Pfanne übriggelassen wurden (z.B. Kartoffelbrei).
- ☐ Speisereste nach Aufbewahrung: Speisereste, die entsorgt werden, nachdem sie im Kühlschrank oder in der Tiefkühltruhe aufbewahrt wurden (z.B. aufbewahrter Kartoffelbrei).

Question 12: Kartoffelprodukte
In Ihrem Haushalt, wie viele Kartoffelprodukte (Pommes / vorgekochte Kartoffeln etc.) wurden in der vergangenen Woche entsorgt?
- Weniger als 10 Pommes / Babykartoffeln / Stücke
- 10 bis 25 Pommes / Baby Kartoffeln / Stücke
- Mehr als 10 Pommes / Baby Kartoffeln / Stücke (entspricht ungefähr einer halben 500g Packung)
- Ganze Packung (750g) Pommes / Baby Kartoffeln / Stücke
- Mehr als eine Packung (750g) Pommes/ Baby Kartoffeln / Stücke

Question 13: Kartoffelprodukte
Zu welcher Kategorie hat die (Mehrheit) der entsorgten Kartoffelprodukte gehört?
Bitte kreuzen Sie die Kategorie an, die am meisten aufgetreten ist. Sie können mehr als ein Kästchen ankreuzen, wenn mehrere Kategorien in der gleichen Menge aufgetreten sind.
- ☐ Komplett unverbrauchte Lebensmittel: Lebensmittel, die entsorgt werden, die überhaupt nicht verwendet wurden (z. B. komplette Packung Pommes).
- ☐ Teilweise verbrauchte Lebensmittel: Lebensmittel, die entsorgt werden, nachdem sie teilweise verbraucht wurden (z.B. halbe Packung Pommes).
- ☐ Speisereste: Lebensmittelreste, die entsorgt werden, nachdem sie auf dem Teller, im Topf oder in der Pfanne übriggelassen wurden.
- ☐ Speisereste nach Aufbewahrung: Speisereste, die entsorgt werden, nachdem sie im Kühlschrank oder in der Tiefkühltruhe aufbewahrt wurden.

Question 14: Nudeln
In Ihrem Haushalt, wie viel Nudeln wurden in der vergangenen Woche entsorgt?
Ein Servierlöffel entspricht 50 g.
- Weniger als ein Servierlöffel
- 1 bis 2 Servierlöffel
- 2 bis 4 Servierlöffel
- 4 bis 6 Servierlöffel
- Mehr als 6 Servierlöffel

Question 15: Nudeln
Zu welcher Kategorie hat die (Mehrheit) der entsorgten Nudeln gehört?
Bitte kreuzen Sie die Kategorie an, die am meisten aufgetreten ist. Sie können mehr als ein Kästchen ankreuzen, wenn mehrere Kategorien in der gleichen Menge aufgetreten sind.
- ☐ Komplett unverbrauchte Lebensmittel: Lebensmittel, die entsorgt werden, die überhaupt nicht verwendet wurden (z. B. komplette Packung Nudeln).
- ☐ Teilweise verbrauchte Lebensmittel: Lebensmittel, die entsorgt werden, nachdem sie teilweise verbraucht wurden (z.B. halbe Packung Nudeln).
- ☐ Speisereste: Lebensmittelreste, die entsorgt werden, nachdem sie auf dem Teller, im Topf oder in der Pfanne übriggelassen wurden.
- ☐ Speisereste nach Aufbewahrung: Speisereste, die entsorgt werden, nachdem sie im Kühlschrank oder in der Tiefkühltruhe aufbewahrt wurden.

Question 16: Reis und anderes Getreide (einschließlich Wraps, Couscous etc.)
In Ihrem Haushalt, wie viel Reis und anderes Getreide (einschließlich Wraps, Couscous etc.) wurden in der vergangenen Woche entsorgt?
Ein Servierlöffel entspricht 50 g.

- Weniger als ein Servierlöffel
- 1 bis 2 Servierlöffel
- 2 bis 4 Servierlöffel
- 4 bis 6 Servierlöffel
- Mehr als 6 Servierlöffel

Question 17: Reis und anderes Getreide (einschließlich Wraps, Couscous etc.)
Zu welcher Kategorie hat die (Mehrheit) des entsorgten Reises gehört? *Bitte kreuzen Sie die Kategorie an, die am meisten aufgetreten ist. Sie können mehr als ein Kästchen ankreuzen, wenn mehrere Kategorien in der gleichen Menge aufgetreten sind.*

- ☐ Komplett unverbrauchte Lebensmittel: Lebensmittel, die entsorgt werden, die überhaupt nicht verwendet wurden (z. B. komplette Packung Reis).
- ☐ Teilweise verbrauchte Lebensmittel: Lebensmittel, die entsorgt werden, nachdem sie teilweise verbraucht wurden (z.B. halbe Packung Reis).
- ☐ Speisereste: Lebensmittelreste, die entsorgt werden, nachdem sie auf dem Teller, im Topf oder in der Pfanne übriggelassen wurden.
- ☐ Speisereste nach Aufbewahrung: Speisereste, die entsorgt werden, nachdem sie im Kühlschrank oder in der Tiefkühltruhe aufbewahrt wurden.

Question 18: Bohnen, Linsen, Kichererbsen
In Ihrem Haushalt, wie viel Bohnen, Linsen, Kichererbsen etc. wurden in der vergangenen Woche entsorgt?
Ein Servierlöffel entspricht 50 g.

- Weniger als ein Servierlöffel
- 1 bis 2 Servierlöffel
- 2 bis 4 Servierlöffel
- 4 bis 6 Servierlöffel
- Mehr als 6 Servierlöffel

Question 19: Bohnen, Linsen, Kichererbsen
Zu welcher Kategorie hat die (Mehrheit) des entsorgten Bohnen gehört?
Bitte kreuzen Sie die Kategorie an, die am meisten aufgetreten ist. Sie können mehr als ein Kästchen ankreuzen, wenn mehrere Kategorien in der gleichen Menge aufgetreten sind.

- ☐ Komplett unverbrauchte Lebensmittel: Lebensmittel, die entsorgt werden, die überhaupt nicht verwendet wurden (z. B. ungeöffnete Dose Bohnen).
- ☐ Teilweise verbrauchte Lebensmittel: Lebensmittel, die entsorgt werden, nachdem sie teilweise verbraucht wurden (z.B. halbe Dose Bohnen).
- ☐ Speisereste: Lebensmittelreste, die entsorgt werden, nachdem sie auf dem Teller, im Topf oder in der Pfanne übriggelassen wurden.
- ☐ Speisereste nach Aufbewahrung: Speisereste, die entsorgt werden, nachdem sie im Kühlschrank oder in der Tiefkühltruhe aufbewahrt wurden.

Question 20: Fleisch
In Ihrem Haushalt, wie viel Fleisch wurden in der vergangenen Woche entsorgt?
Eine Portion bezieht sich auf eine Hähnchenbrust, ein etc. Im Fall von kleineren Stücken, wie Hackfleisch, versuchen Sie die Menge in ganzen Stücken abzuschätzen (z.B. eine Packung Hackfleisch entspricht zwei Portionen).

- Ungefähr eine halbe Portion oder weniger
- Ungefähr eine Portion
- 2 oder 3 Portionen
- 4 oder 5 Portionen
- Mehr als 5 Portionen

Question 21: Fleisch
Zu welcher Kategorie hat die (Mehrheit) des entsorgten Fleisches gehört? *Bitte kreuzen Sie die Kategorie an, die am meisten aufgetreten ist. Sie können mehr als ein Kästchen ankreuzen, wenn mehrere Kategorien in der gleichen Menge aufgetreten sind.*

- ☐ Komplett unverbrauchte Lebensmittel: Lebensmittel, die entsorgt werden, die überhaupt nicht verwendet wurden (z. B. komplette Packung Hackfleisch).
- ☐ Teilweise verbrauchte Lebensmittel: Lebensmittel, die entsorgt werden, nachdem sie teilweise verbraucht wurden (z.B. halbe Packung Hackfleisch).
- ☐ Speisereste: Lebensmittelreste, die entsorgt werden, nachdem sie auf dem Teller, im Topf oder in der Pfanne übriggelassen wurden.
- ☐ Speisereste nach Aufbewahrung: Speisereste, die entsorgt werden, nachdem sie im Kühlschrank oder in der Tiefkühltruhe aufbewahrt wurden.

Question 22: Fleischersatzprodukte
In Ihrem Haushalt, wie viel Fleischersatzprodukte wurden in der vergangenen Woche entsorgt?
Eine Portion bezieht sich auf eine vegetarische Burger. Im Fall von kleineren Stücken, wie vegetarische Hackfleisch, versuchen Sie die Menge in ganzen Stücken abzuschätzen (z.B. eine Packung vegetarische Hackfleisch entspricht zwei Portionen).

- o Ungefähr eine halbe Portion oder weniger
- o Ungefähr eine Portion
- o 2 oder 3 Portionen
- o 4 oder 5 Portionen
- o Mehr als 5 Portionen

Question 23: Fleischersatzprodukte
Zu welcher Kategorie hat die (Mehrheit) des entsorgten Fleischersatzprodukte gehört?
Bitte kreuzen Sie die Kategorie an, die am meisten aufgetreten ist. Sie können mehr als ein Kästchen ankreuzen, wenn mehrere Kategorien in der gleichen Menge aufgetreten sind.

- ☐ Komplett unverbrauchte Lebensmittel: Lebensmittel, die entsorgt werden, die überhaupt nicht verwendet wurden (z. B. komplette Packung vegetarische Hackfleisch).
- ☐ Teilweise verbrauchte Lebensmittel: Lebensmittel, die entsorgt werden, nachdem sie teilweise verbraucht wurden (z.B. halbe Packung vegetarische Hackfleisch).
- ☐ Speisereste: Lebensmittelreste, die entsorgt werden, nachdem sie auf dem Teller, im Topf oder in der Pfanne übriggelassen wurden.
- ☐ Speisereste nach Aufbewahrung: Speisereste, die entsorgt werden, nachdem sie im Kühlschrank oder in der Tiefkühltruhe aufbewahrt wurden.

Question 24: Fisch
In Ihrem Haushalt, wie viel Fisch wurde in der vergangenen Woche entsorgt? *Eine Portion entspricht einem filletierten Fisch, einem Stück Lachs, etc.*

- o Ungefähr eine halbe Portion oder weniger
- o Ungefähr eine Portion
- o 2 oder 3 Portionen
- o 4 oder 5 Portionen
- o Mehr als 5 Portionen

Question 25: Fisches
Zu welcher Kategorie hat die (Mehrheit) des entsorgten Fisches gehört?
Bitte kreuzen Sie die Kategorie an, die am meisten aufgetreten ist. Sie können mehr als ein Kästchen ankreuzen, wenn mehrere Kategorien in der gleichen Menge aufgetreten sind.

- ☐ Komplett unverbrauchte Lebensmittel: Lebensmittel, die entsorgt werden, die überhaupt nicht verwendet wurden (z. B. komplette Packung Fisch).
- ☐ Teilweise verbrauchte Lebensmittel: Lebensmittel, die entsorgt werden, nachdem sie teilweise verbraucht wurden (z.B. halbe Packung Fisch).
- ☐ Speisereste: Lebensmittelreste, die entsorgt werden, nachdem sie auf dem Teller, im Topf oder in der Pfanne übriggelassen wurden.
- ☐ Speisereste nach Aufbewahrung: Speisereste, die entsorgt werden, nachdem sie im Kühlschrank oder in der Tiefkühltruhe aufbewahrt wurden.

Question 26: Brotbelags
In Ihrem Haushalt, wie viel Brotbelag wurde in der vergangenen Woche entsorgt?
Eine Portion entspricht der verwendeten Menge auf einer Scheibe Brot / Sandwich / einer Portion Baguette.
- o Ungefähr eine halbe Portion oder weniger
- o Ungefähr eine Portion
- o 2 oder 3 Portionen
- o 4 oder 5 Portionen
- o Mehr als 5 Portionen

Question 27: Brotbelags
Zu welcher Kategorie hat die (Mehrheit) des entsorgten Brotbelags gehört?
Bitte kreuzen Sie die Kategorie an, die am meisten aufgetreten ist. Sie können mehr als ein Kästchen ankreuzen, wenn mehrere Kategorien in der gleichen Menge aufgetreten sind.
- ☐ Komplett unverbrauchte Lebensmittel: Lebensmittel, die entsorgt werden, die überhaupt nicht verwendet wurden (z. B. komplette Packung Wurstscheiben).
- ☐ Teilweise verbrauchte Lebensmittel: Lebensmittel, die entsorgt werden, nachdem sie teilweise verbraucht wurden (z.B. halbe Packung Wurstscheiben).
- ☐ Speisereste: Lebensmittelreste, die entsorgt werden, nachdem sie auf dem Teller, im Topf oder in der Pfanne übriggelassen wurden.
- ☐ Speisereste nach Aufbewahrung: Speisereste, die entsorgt werden, nachdem sie im Kühlschrank oder in der Tiefkühltruhe aufbewahrt wurden.

Question 28: Brots
In Ihrem Haushalt, wie viel Brot wurde in der vergangenen Woche entsorgt?
Ein Brötchen, eine Portion Baguette oder ein Sandwich zählen als eine Scheibe Brot.
- o Weniger als eine Scheibe
- o Eine oder zwei Scheiben
- o Ungefähr ein halber Laib
- o Ungefähr ein ganzer Laib
- o Mehr als ein Laib

Question 29: Brots
Zu welcher Kategorie hat die (Mehrheit) des entsorgten Brots gehört?
Bitte kreuzen Sie die Kategorie an, die am meisten aufgetreten ist. Sie können mehr als ein Kästchen ankreuzen, wenn mehrere Kategorien in der gleichen Menge aufgetreten sind.
- ☐ Komplett unverbrauchte Lebensmittel: Lebensmittel, die entsorgt werden, die überhaupt nicht verwendet wurden (z. B. ganzer Laib Brot).
- ☐ Teilweise verbrauchte Lebensmittel: Lebensmittel, die entsorgt werden, nachdem sie teilweise verbraucht wurden (z.B. einzelne Brotscheiben).
- ☐ Speisereste: Lebensmittelreste, die entsorgt werden, nachdem sie auf dem Teller, im Topf oder in der Pfanne übriggelassen wurden (z.B. Brotkrusten).
- ☐ Speisereste nach Aufbewahrung: Speisereste, die entsorgt werden, nachdem sie im Kühlschrank oder in der Tiefkühltruhe aufbewahrt wurden.

Question 30: Müslis
In Ihrem Haushalt, wie viel Müsli wurde in der vergangenen Woche entsorgt? *Eine Portion entspricht der Menge einer Frühstücksschale.*
- o Weniger als eine halbe Portion
- o Eine halbe bis eineinhalb Portionen
- o Mehrere Portionen (ungefähr eine halbe Packung)
- o Ungefähr eine ganze Packung
- o Mehrere Packungen

Question 31: Müslis
Zu welcher Kategorie hat die (Mehrheit) des entsorgten Müslis gehört?
Bitte kreuzen Sie die Kategorie an, die am meisten aufgetreten ist. Sie können mehr als ein Kästchen ankreuzen, wenn mehrere Kategorien in der gleichen Menge aufgetreten sind.
- ☐ Komplett unverbrauchte Lebensmittel: Lebensmittel, die entsorgt werden, die überhaupt nicht verwendet wurden (z. B. komplette Packung Müsli).
- ☐ Teilweise verbrauchte Lebensmittel: Lebensmittel, die entsorgt werden, nachdem sie teilweise verbraucht wurden (z.B. halbe Packung Müsli).
- ☐ Speisereste: Lebensmittelreste, die entsorgt werden, nachdem sie auf dem Teller, im Topf oder in der Pfanne übriggelassen wurden.
- ☐ Speisereste nach Aufbewahrung: Speisereste, die entsorgt werden, nachdem sie im Kühlschrank oder in der Tiefkühltruhe aufbewahrt wurden.

Question 32: Joghurts
In Ihrem Haushalt, wie viel Joghurt, Pudding, etc. wurde in der vergangenen Woche entsorgt? *Eine Portion entspricht der Menge einer kleinen Schale.*

- o Weniger als eine halbe Portion
- o Eine halbe bis eineinhalb Portionen
- o Mehrere Portionen (ungefähr eine halbe Literpackung)
- o Ungefähr eine ganze Literpackung
- o Mehrere Literpackungen

Question 33: Joghurts
Zu welcher Kategorie hat die (Mehrheit) des entsorgten Joghurts, Puddings, etc. gehört?
Bitte kreuzen Sie die Kategorie an, die am meisten aufgetreten ist. Sie können mehr als ein Kästchen ankreuzen, wenn mehrere Kategorien in der gleichen Menge aufgetreten sind.

- ☐ Komplett unverbrauchte Lebensmittel: Lebensmittel, die entsorgt werden, die überhaupt nicht verwendet wurden (z. B. komplette Packung Joghurt).
- ☐ Teilweise verbrauchte Lebensmittel: Lebensmittel, die entsorgt werden, nachdem sie teilweise verbraucht wurden (z.B. halbe Packung Joghurt).
- ☐ Speisereste: Lebensmittelreste, die entsorgt werden, nachdem sie auf dem Teller, im Topf oder in der Pfanne übriggelassen wurden.
- ☐ Speisereste nach Aufbewahrung: Speisereste, die entsorgt werden, nachdem sie im Kühlschrank oder in der Tiefkühltruhe aufbewahrt wurden.

Question 34: Käses
In Ihrem Haushalt, wie viel Käse (Käsewürfel, Käse am Stück, Streukäse; nicht: Käse als Brotbelag) wurde in der vergangenen Woche entsorgt? *Eine Handvoll Käse zählt als ein Stück.*

- o Weniger als ein Stück
- o Ungefähr ein Stück
- o 1 bis 3 Stücke
- o 4 bis 5 Stücke
- o Mehr als 5 Stücke

NOTE with question 34: Due to progressive insights, increasing the measure options can be considered, as 5 cheese dices is low compared to the potential maximum gram of waste of the other categories. However, please keep in mind that this will change the set-up slightly, when comparing the results with the earlier applications of this survey.

Question 35: Käses
Zu welcher Kategorie hat die (Mehrheit) des entsorgten Käses gehört?
Bitte kreuzen Sie die Kategorie an, die am meisten aufgetreten ist. Sie können mehr als ein Kästchen ankreuzen, wenn mehrere Kategorien in der gleichen Menge aufgetreten sind.

- ☐ Komplett unverbrauchte Lebensmittel: Lebensmittel, die entsorgt werden, die überhaupt nicht verwendet wurden (z. B. kompletter Käse am Stück).
- ☐ Teilweise verbrauchte Lebensmittel: Lebensmittel, die entsorgt werden, nachdem sie teilweise verbraucht wurden (z.B. angefangener Käse am Stück).
- ☐ Speisereste: Lebensmittelreste, die entsorgt werden, nachdem sie auf dem Teller, im Topf oder in der Pfanne übriggelassen wurden.
- ☐ Speisereste nach Aufbewahrung: Speisereste, die entsorgt werden, nachdem sie im Kühlschrank oder in der Tiefkühltruhe aufbewahrt wurden.

Question 36: Eier
In Ihrem Haushalt, wie viele Eier wurden in der vergangenen Woche entsorgt?

- o Weniger als 1 Ei
- o 1 Ei
- o 2 bis 3 Eier
- o 4 bis 5 Eier
- o Mehr als 5 Eier

Question 37: Eier
Zu welcher Kategorie hat die (Mehrheit) der entsorgten Eier gehört?
Bitte kreuzen Sie die Kategorie an, die am meisten aufgetreten ist. Sie können mehr als ein Kästchen ankreuzen, wenn mehrere Kategorien in der gleichen Menge aufgetreten sind.
- ☐ Komplett unverbrauchte Lebensmittel: Lebensmittel, die entsorgt werden, die überhaupt nicht verwendet wurden (z. B. komplette Eier).
- ☐ Teilweise verbrauchte Lebensmittel: Lebensmittel, die entsorgt werden, nachdem sie teilweise verbraucht wurden (z.B. Eiweiß).
- ☐ Speisereste: Lebensmittelreste, die entsorgt werden, nachdem sie auf dem Teller, im Topf oder in der Pfanne übriggelassen wurden.
- ☐ Speisereste nach Aufbewahrung: Speisereste, die entsorgt werden, nachdem sie im Kühlschrank oder in der Tiefkühltruhe aufbewahrt wurden.

Question 38: Suppe und Eintöpfe
In Ihrem Haushalt, wie viel Suppe und Eintöpfe wurde in der vergangenen Woche entsorgt?
- o Weniger als eine halbe Schöpfkelle
- o Eine halbe bis eineinhalb Schöpfkellen
- o Mehrere Schöpfkellen (ungefähr ein halber Liter)
- o Ungefähr ein Liter
- o Mehr als ein Liter

Question 39: Suppe und Eintöpfe
Zu welcher Kategorie hat die (Mehrheit) der entsorgten Suppe und Eintöpfe gehört?
Bitte kreuzen Sie die Kategorie an, die am meisten aufgetreten ist. Sie können mehr als ein Kästchen ankreuzen, wenn mehrere Kategorien in der gleichen Menge aufgetreten sind.
- ☐ Komplett unverbrauchte Lebensmittel: Lebensmittel, die entsorgt werden, die überhaupt nicht verwendet wurden (z. B. komplette Packung Suppe).
- ☐ Teilweise verbrauchte Lebensmittel: Lebensmittel, die entsorgt werden, nachdem sie teilweise verbraucht wurden (z.B. halbe Packung Suppe).
- ☐ Speisereste: Lebensmittelreste, die entsorgt werden, nachdem sie auf dem Teller, im Topf oder in der Pfanne übriggelassen wurden.
- ☐ Speisereste nach Aufbewahrung: Speisereste, die entsorgt werden, nachdem sie im Kühlschrank oder in der Tiefkühltruhe aufbewahrt wurden.

Question 40: Soßen
In Ihrem Haushalt, wie viel Soßen (Ketchup, Mayonnaise, Cocktailsoße, etc.) wurden in der vergangenen Woche entsorgt? Ein Esslöffel = 20 gram
- o Weniger als ein Esslöffel
- o 1 bis 3 Esslöffel
- o Mehrere Esslöffel (ungefähr ein halbes Glas / Flasche)
- o Ungefähr ein ganzes Glas / Flasche
- o Mehr als ein Glas / Flasche

Question 41: Soßen
Zu welcher Kategorie hat die (Mehrheit) der entsorgten Soßen gehört?
Bitte kreuzen Sie die Kategorie an, die am meisten aufgetreten ist. Sie können mehr als ein Kästchen ankreuzen, wenn mehrere Kategorien in der gleichen Menge aufgetreten sind.
- ☐ Komplett unverbrauchte Lebensmittel: Lebensmittel, die entsorgt werden, die überhaupt nicht verwendet wurden (z. B. komplette Flasche Soße).
- ☐ Teilweise verbrauchte Lebensmittel: Lebensmittel, die entsorgt werden, nachdem sie teilweise verbraucht wurden (z.B. halbe Flasche Soße).
- ☐ Speisereste: Lebensmittelreste, die entsorgt werden, nachdem sie auf dem Teller, im Topf oder in der Pfanne übriggelassen wurden.
- ☐ Speisereste nach Aufbewahrung: Speisereste, die entsorgt werden, nachdem sie im Kühlschrank oder in der Tiefkühltruhe aufbewahrt wurden.

Question 42: Süßigkeiten
In Ihrem Haushalt, wie viele Süßigkeiten / Kekse / Müsliriegel / Schokoriegel wurden in der vergangenen Woche entsorgt? *Eine Portion entspricht einer Handvoll Gummibärchen, einem kleinen Schokoriegel, einem Keks, etc.)*

- o Ungefähr eine halbe Portion oder weniger
- o Ungefähr eine Portion
- o 2 oder 3 Portionen
- o 4 oder 5 Portionen
- o Mehr als 5 Portionen

Question 43: Süßigkeiten
Zu welcher Kategorie hat die (Mehrheit) der entsorgten Süßigkeiten gehört?
Bitte kreuzen Sie die Kategorie an, die am meisten aufgetreten ist. Sie können mehr als ein Kästchen ankreuzen, wenn mehrere Kategorien in der gleichen Menge aufgetreten sind.

- ☐ Komplett unverbrauchte Lebensmittel: Lebensmittel, die entsorgt werden, die überhaupt nicht verwendet wurden (z. B. komplette Packung Kekse).
- ☐ Teilweise verbrauchte Lebensmittel: Lebensmittel, die entsorgt werden, nachdem sie teilweise verbraucht wurden (z.B. halbe Packung Kekse).
- ☐ Speisereste: Lebensmittelreste, die entsorgt werden, nachdem sie auf dem Teller, im Topf oder in der Pfanne übriggelassen wurden.
- ☐ Speisereste nach Aufbewahrung: Speisereste, die entsorgt werden, nachdem sie im Kühlschrank oder in der Tiefkühltruhe aufbewahrt wurden.

Question 44: Cracker / Nüsse
In Ihrem Haushalt, wie viele Cracker / Nüsse wurden in der vergangenen Woche entsorgt? *Eine Portion entspricht einer Handvoll Cracker / Nüsse.)*

- o Ungefähr eine halbe Portion oder weniger
- o Ungefähr eine Portion
- o 2 oder 3 Portionen
- o 4 oder 5 Portionen
- o Mehr als 5 Portionen

Question 45: Cracker / Nüsse
Zu welcher Kategorie hat die (Mehrheit) der entsorgten Cracker / Nüsse gehört?
Bitte kreuzen Sie die Kategorie an, die am meisten aufgetreten ist. Sie können mehr als ein Kästchen ankreuzen, wenn mehrere Kategorien in der gleichen Menge aufgetreten sind.

- ☐ Komplett unverbrauchte Lebensmittel: Lebensmittel, die entsorgt werden, die überhaupt nicht verwendet wurden (z. B. komplette Packung Cracker).
- ☐ Teilweise verbrauchte Lebensmittel: Lebensmittel, die entsorgt werden, nachdem sie teilweise verbraucht wurden (z.B. halbe Packung Cracker).
- ☐ Speisereste: Lebensmittelreste, die entsorgt werden, nachdem sie auf dem Teller, im Topf oder in der Pfanne übriggelassen wurden.
- ☐ Speisereste nach Aufbewahrung: Speisereste, die entsorgt werden, nachdem sie im Kühlschrank oder in der Tiefkühltruhe aufbewahrt wurden.

Question 46: Nicht- alkoholischen Getränke
In Ihrem Haushalt, wie viele nicht- alkoholische Getränke (Milch, Saft, Softdrinks; nicht: Wasser, Tee, Kaffee, Sirup) wurden in der vergangenen Woche entsorgt?

- o Weniger als ein halbes Glas
- o Ein halbes bis eineinhalb Gläser
- o Mehrere Gläser (ungefähr ein halber Liter)
- o Ungefähr ein Liter
- o Mehr als ein Liter

Question 47: Nicht- alkoholischen Getränke
Zu welcher Kategorie hat die (Mehrheit) der entsorgten nicht- alkoholischen Getränke gehört?
Bitte kreuzen Sie die Kategorie an, die am meisten aufgetreten ist. Sie können mehr als ein Kästchen ankreuzen, wenn mehrere Kategorien in der gleichen Menge aufgetreten sind.

- ☐ Komplett unverbrauchte Lebensmittel: Lebensmittel, die entsorgt werden, die überhaupt nicht verwendet wurden (z. B. komplette Packung Milch).
- ☐ Teilweise verbrauchte Lebensmittel: Lebensmittel, die entsorgt werden, nachdem sie teilweise verbraucht wurden (z.B. halbe Packung Milch).
- ☐ Speisereste: Lebensmittelreste, die entsorgt werden, nachdem sie im Glas übriggelassen wurden.
- ☐ Speisereste nach Aufbewahrung: Speisereste, die entsorgt werden, nachdem sie aufbewahrt wurden.

Question 48: Alkoholischen Getränke
In Ihrem Haushalt, wie viele alkoholische Getränke wurden in der vergangenen Woche entsorgt?

- o Weniger als ein halbes Glas
- o Ein halbes bis eineinhalb Gläser
- o Mehrere Gläser (ungefähr ein halber Liter)
- o Ungefähr ein Liter
- o Mehr als ein Liter

Question 49: Alkoholischen Getränke

Zu welcher Kategorie hat die (Mehrheit) der entsorgten alkoholischen Getränke gehört? *Bitte kreuzen Sie die Kategorie an, die am meisten aufgetreten ist. Sie können mehr als ein Kästchen ankreuzen, wenn mehrere Kategorien in der gleichen Menge aufgetreten sind.*

- ☐ Komplett unverbrauchte Lebensmittel: Lebensmittel, die entsorgt werden, die überhaupt nicht verwendet wurden (z. B. eine Flasche Wein).
- ☐ Teilweise verbrauchte Lebensmittel: Lebensmittel, die entsorgt werden, nachdem sie teilweise verbraucht wurden (z.B. halbe Flasche Wein).
- ☐ Speisereste: Lebensmittelreste, die entsorgt werden, nachdem sie im Glas übriggelassen wurden.
- ☐ Speisereste nach Aufbewahrung: Speisereste, die entsorgt werden, nachdem sie aufbewahrt wurden.

Appendix D: Household Food Waste Questionnaire in Hungarian

Introduction

Múlt héten küldtünk Önnek egy emailt, amiben arra kértük, hogy alaposan figyelje meg, milyen és mennyi élelmiszert és italt dobnak ki a háztartásában. Az alábbi kérdőív ezekről a termékekről szól. Emlékeztetőül:

Jelen kutatásban kidobott élelmiszernek számít:

- Minden olyan élelmiszer és ital, amit interneten vagy boltban vásároltak vagy otthon termesztettek, és amelyeket kidobtak.
- Ebbe beletartoznak azok a termékek is, amelyek megromlottak vagy lejártak és emiatt kellett őket kidobni.
- Nem számít, hogy a kidobott élelmiszer hová került: rendes szemetesbe, szelektív hulladékgyűjtőbe vagy komposztálóba, vagy háziállatnak (kutya, macska, madár stb.) lett odaadva – ezek mind kidobásnak számítanak.

Jelen kutatásban **nem** számítanak kidobott élelmiszernek, ezért a kérdések nem vonatkoznak a következőkre:

- Csontok, zöldség- vagy gyümölcshéjak, -magok, csutkák.
- Étteremben vagy menzán megmaradt és kidobott ételek és italok.

Questionnaire

Question 1: Válassza ki azokat az élelmiszereket, amiket az elmúlt egy hétben ki kellett dobni. Főtt ételek esetén a fő összetevőket válassza ki.

☐ Friss zöldség és saláta
☐ Nem friss zöldség (üveges / konzerv / fagyasztott)
☐ Friss gyümölcs
☐ Nem friss gyümölcs (befőtt / konzerv / szárított / fagyasztott)
☐ Burgonya
☐ Burgonya alapú ételek (hasábburgonya, előfőzött/fagyasztott krumpli stb.)
☐ Tészta
☐ Rizs és egyéb köret (lepény, kuszkusz, bulgur stb., kivéve burgonya)
☐ Bab, lencse, csicseriborsó stb.
☐ Hús (kivéve: felvágott)
☐ Húshelyettesítő
☐ Hal
☐ Kenyérfeltétek (felvágott, sajt, vaj, édes krém stb.)
☐ Kenyér, zsemle, kifli stb.
☐ Gabonapelyhek (müzli, zabpehely, zabkása, granola stb.)
☐ Joghurt, tejföl, túró stb.
☐ Sajt (tömbsajt, kockasajt, sajtkrém, kivéve: sajt kenyérfeltétként)
☐ Tojás
☐ Leves, főzelék
☐ Szószok (ketchup, majonéz, mustár stb.)
☐ Édesség, sütemény, keksz, müzliszelet, csokoládé
☐ Chips, ropik, magvak
☐ Alkoholmentes italok (tej*, gyümölcslé, szénsavas üdítő. Kivéve: víz, tea, kávé, higított szörp)
☐ Alkoholos italok
☐ Egyáltalán nem dobtam ki ételt vagy italt

* *NOTE*: Due to progressive insights, creating a separate category for "Milk" can be considered, as this is a frequently disposed of food product. However, please keep in mind that this will change the set-up slightly, when comparing the results with the earlier applications of this survey.

Introduction to the next part of the questionnaire

A kidobott élelmiszerek több csoportra oszthatóak, az alábbiak szerint. Kérjük, figyelmesen olvassa el ezt a részt, mert ezeket a kategóriákat használjuk a kérdésekben is.

1) <u>Hiánytalan/bontatlan élelmiszerek:</u> olyan kidobott élelmiszer, amely egyáltalán nem lett felhasználva. Például bontatlan csomagolású élelmiszerek, több egységet tartalmazó csomagok bontatlan részei, egész csomag alma, egész kenyér.
2) <u>Felbontott/megkezdett élelmiszerek:</u>olyan élelmiszerek, amelyekből valamennyi fogyott, de aztán kidobásra kerültek. Például néhány szelet kenyér, fél csomag felvágott, fél fej hagyma, fél doboz tej.
3) <u>Ételmaradékok:</u> tányéron, tálban, serpenyőben hagyott étel, ami kidobásra került. Például krumplipüré, főtt rizs, ami nem fogyott el, megmaradt szendvics.
4) <u>Tárolt ételmaradékok:</u> későbbi fogyasztási céllal hűtőben vagy fagyasztóban tárolt ételmaradékok, amelyek végül a szemetesben végezték. Például egy adag múlt heti, lefagyasztott tésztaétel.

A következőkben az elmúlt hét folyamán az Ön háztartásában kidobott, különböző típusú ételekkel és italokkal kapcsolatban teszünk fel kérdéseket. Először arra leszünk kíváncsiak, hogy bizonyos fajta ételekből mekkora mennyiség került a szemetesbe a háztartásában az elmúlt hét folyamán. Ezután arra kérjük, hogy válassza ki, a kidobott ételek többsége melyik kategóriába tartozik (bontatlan, megkezdett, ételmaradék, tárolt ételmaradék). Kérjük, legyen figyelmes, hogy a kérdés melyik fajta élelmiszerre vonatkozik.

NOTE: The next questions are only shown if respondents indicated waste in the respective category in Question 1

Question 2: Friss zöldségek és saláták
Mekkora mennyiségű hulladék keletkezett a háztartásában friss zöldségekből és salátából az elmúlt hét folyamán? Egy adag = fél merőkanál, kb. 50 gramm. Összehasonlításképp: ez fél póréhagyma vagy 4 darab gomba.

- Kevesebb, mint egy adag
- 1-2 adag
- 2-4 adag
- 4-6 adag
- Több, mint 6 adag

Question 3: Friss zöldségek és saláták
Melyik kategóriába sorolható a kidobott friss zöldségek és saláták nagy része? *Kérjük, válassza ki azt a kategóriát, amelyikből a legtöbb volt. Többet is választhat, ha több kategóriában ugyanannyi hulladék keletkezett.*

- ☐ Hiánytalan/bontatlan élelmiszerek: olyan kidobott élelmiszer, amely egyáltalán nem lett felhasználva (pl. teljes fej káposzta)
- ☐ Felbontott/megkezdett élelmiszerek: olyan élelmiszerek, amelyekből valamennyi fogyott, de aztán kidobásra kerültek (pl. fél fej hagyma)
- ☐ Ételmaradékok: tányéron, tálban, serpenyőben hagyott étel, ami kidobásra került.
- ☐ Tárolt ételmaradékok: későbbi fogyasztási céllal hűtőben vagy fagyasztóban tárolt ételmaradékok, amelyek végül a szemetesben végezték.

Question 4: Nem friss zöldség
Mekkora mennyiségű hulladék keletkezett a háztartásában nem friss zöldségekből (üveges / konzerv / fagyasztott) az elmúlt hét folyamán? Egy adag = fél merőkanál, kb. 50 gramm. Összehasonlításképp: ez fél póréhagyma vagy 4 darab gomba.

- Kevesebb, mint egy adag
- 1-2 adag
- 2-4 adag
- 4-6 adag
- Több, mint 6 adag

Question 5: Nem friss zöldség
Melyik kategóriába sorolható a kidobott nem friss zöldségek nagy része? *Kérjük, válassza ki azt a kategóriát, amelyikből a legtöbb volt. Többet is választhat, ha több kategóriában ugyanannyi hulladék keletkezett.*

- ☐ Hiánytalan/bontatlan élelmiszerek: olyan kidobott élelmiszer, amely egyáltalán nem lett felhasználva (pl. bontatlan fagyasztott/konzerv spenót)
- ☐ Felbontott/megkezdett élelmiszerek: olyan élelmiszerek, amelyekből valamennyi fogyott, de aztán kidobásra kerültek (pl. bontott konzerv kukorica)
- ☐ Ételmaradékok: tányéron, tálban, serpenyőben hagyott étel, ami kidobásra került.
- ☐ Tárolt ételmaradékok: későbbi fogyasztási céllal hűtőben vagy fagyasztóban tárolt ételmaradékok, amelyek végül a szemetesben végezték.

Question 6: Friss gyümölcsök
Mekkora mennyiségű hulladék keletkezett a háztartásában friss gyümölcsökből az elmúlt hét folyamán?
Egy alma vagy egy banán egy darab gyümölcsnek számít. A kisebb méretű gyümölcsökből (pl. eper, szőlő) egy tálkányi számít egy darabnak.

- Körülbelül negyed darab gyümölcs vagy kevesebb
- Körülbelül fél darab gyümölcs
- Körülbelül egy darab gyümölcs
- 2-4 darab gyümölcs
- Több mint 4 darab gyümölcs

Question 7: Friss gyümölcsök
Melyik kategóriába sorolható a kidobott friss gyümölcsök nagy része? *Kérjük, válassza ki azt a kategóriát, amelyikből a legtöbb volt. Többet is választhat, ha több kategóriában ugyanannyi hulladék keletkezett.*

- ☐ Hiánytalan/bontatlan élelmiszerek: olyan kidobott élelmiszer, amely egyáltalán nem lett felhasználva (pl. egy alma)
- ☐ Felbontott/megkezdett élelmiszerek: olyan élelmiszerek, amelyekből valamennyi fogyott, de aztán kidobásra kerültek (pl. fél alma, ami nem lett megfőzve vagy megsütve)
- ☐ Ételmaradékok: tányéron, tálban, serpenyőben hagyott étel, ami kidobásra került (félig megevett alma vagy gyümölcssaláta)
- ☐ Tárolt ételmaradékok: későbbi fogyasztási céllal hűtőben vagy fagyasztóban tárolt ételmaradékok, amelyek végül a szemetesben végezték (pl. gyümölcssaláta tárolás után)

Question 8: Nem friss gyümölcs
Mekkora mennyiségű hulladék keletkezett a háztartásában nem friss gyümölcsökből (befőtt / konzerv / szárított / fagyasztott) az elmúlt hét folyamán? Egy körte vagy egy őszibarack a konzervből egy darab gyümölcsnek számít. A kisebb méretű gyümölcsökből (pl. málna, áfonya) egy tálkányi számít egy darabnak.

- Körülbelül negyed darab gyümölcs vagy kevesebb
- Körülbelül fél darab gyümölcs
- Körülbelül egy darab gyümölcs
- 2-4 darab gyümölcs
- Több mint 4 darab gyümölcs

Question 9: Nem friss gyümölcs
Melyik kategóriába sorolható a kidobott nem friss gyümölcsök nagy része? *Kérjük, válassza ki azt a kategóriát, amelyikből a legtöbb volt. Többet is választhat, ha több kategóriában ugyanannyi hulladék keletkezett.*

- ☐ Hiánytalan/bontatlan élelmiszerek: olyan kidobott élelmiszer, amely egyáltalán nem lett felhasználva (pl. egy bontatlan gyümölcskonzerv)
- ☐ Felbontott/megkezdett élelmiszerek: olyan élelmiszerek, amelyekből valamennyi fogyott, de aztán kidobásra kerültek (pl. fél doboz gyümölcskonzerv)
- ☐ Ételmaradékok: tányéron, tálban, serpenyőben hagyott étel, ami kidobásra került (pl. gyümölcstál)
- ☐ Tárolt ételmaradékok: későbbi fogyasztási céllal hűtőben vagy fagyasztóban tárolt ételmaradékok, amelyek végül a szemetesben végezték (pl. gyümölcssaláta tárolás után)

Question 10: Burgonya
Mekkora mennyiségű hulladék keletkezett a háztartásában burgonyából az elmúlt hét folyamán?
Egy adag = fél merőkanál, kb. 50 gramm. Összehasonlításképp: ez fél darab közepes méretű krumpli.’

- Kevesebb, mint egy adag
- 1-2 adag
- 2-4 adag
- 4-6 adag
- Több, mint 6 adag

Question 11: Burgonya
Melyik kategóriába sorolható a kidobott burgonya nagy része? *Kérjük, válassza ki azt a kategóriát, amelyikből a legtöbb volt. Többet is választhat, ha több kategóriában ugyanannyi hulladék keletkezett.*

- ☐ Hiánytalan/bontatlan élelmiszerek: olyan kidobott élelmiszer, amely egyáltalán nem lett felhasználva (pl. egy teljes zacskó burgonya)
- ☐ Felbontott/megkezdett élelmiszerek: olyan élelmiszerek, amelyekből valamennyi fogyott, de aztán kidobásra kerültek (pl. fél zacskó burgonya)
- ☐ Ételmaradékok: tányéron, tálban, serpenyőben hagyott étel, ami kidobásra került (pl. burgonyapüré)
- ☐ Tárolt ételmaradékok: későbbi fogyasztási céllal hűtőben vagy fagyasztóban tárolt ételmaradékok, amelyek végül a szemetesben végezték (pl. burgonyapüré tárolás után)

Question 12: Burgonya alapú ételek (hasábburgonya, előfőzött /fagyasztott krumpli stb.
Mekkora mennyiségű hulladék keletkezett a háztartásában burgonya alapú ételekből (hasábburgonya, előfőzött/fagyasztott krumpli stb.) az elmúlt hét folyamán?

- Kevesebb, mint 10 db hasábburgonya / krumplidarab
- 10-25 db hasábburgonya / krumplidarab
- Több, mint 25 db hasábburgonya / krumplidarab (körülbelül egy fél kilós csomag fele)
- 1 csomag (750 g) hasábburgonya / krumpli
- Több, mint 1 csomag (750 g) hasábburgonya / krumpli

Question 13: Burgonya alapú ételek (hasábburgonya, előfőzött/fagyaasztott krumpli stb.
Melyik kategóriába sorolható a kidobott burgonya alapú félkész ételek nagy része? *Kérjük, válassza ki azt a kategóriát, amelyikből a legtöbb volt. Többet is választhat, ha több kategóriában ugyanannyi hulladék keletkezett.*

- ☐ Hiánytalan/bontatlan élelmiszerek: olyan kidobott élelmiszer, amely egyáltalán nem lett felhasználva (pl. egy teljes csomag hasábburgonya)
- ☐ Felbontott/megkezdett élelmiszerek: olyan élelmiszerek, amelyekből valamennyi fogyott, de aztán kidobásra kerültek (pl. fél csomag hasábburgonya)
- ☐ Ételmaradékok: tányéron, tálban, serpenyőben hagyott étel, ami kidobásra került.
- ☐ Tárolt ételmaradékok: későbbi fogyasztási céllal hűtőben vagy fagyasztóban tárolt ételmaradékok, amelyek végül a szemetesben végezték.

Question 14: Tészta
Mekkora mennyiségű hulladék keletkezett a háztartásában tésztából az elmúlt hét folyamán?
Egy adag = fél merőkanál, kb. 50 gramm.

- Kevesebb, mint egy adag
- 1-2 adag
- 2-4 adag
- 4-6 adag
- Több, mint 6 adag

Question 15: Tészta
Melyik kategóriába sorolható a kidobott tészta nagy része? *Kérjük, válassza ki azt a kategóriát, amelyikből a legtöbb volt. Többet is választhat, ha több kategóriában ugyanannyi hulladék keletkezett.*

- ☐ Hiánytalan/bontatlan élelmiszerek: olyan kidobott élelmiszer, amely egyáltalán nem lett felhasználva (pl. egy teljes csomag tészta)
- ☐ Felbontott/megkezdett élelmiszerek: olyan élelmiszerek, amelyekből valamennyi fogyott, de aztán kidobásra kerültek (pl. fél csomag tészta)
- ☐ Ételmaradékok: tányéron, tálban, serpenyőben hagyott étel, ami kidobásra került.
- ☐ Tárolt ételmaradékok: későbbi fogyasztási céllal hűtőben vagy fagyasztóban tárolt ételmaradékok, amelyek végül a szemetesben végezték.

Question 16: Rizs és egyéb köret (lepény, kuszkusz, bulgur stb., kivéve: burgonya
Mekkora mennyiségű hulladék keletkezett a háztartásában rizsből és egyéb köretből az elmúlt hét folyamán?
Egy adag = fél merőkanál, kb. 50 gramm.

- Kevesebb, mint egy adag
- 1-2 adag
- 2-4 adag
- 4-6 adag
- Több, mint 6 adag

Question 17: Rizs és egyéb köret (lepény, kuszkusz, bulgur stb., kivéve: burgonya
Melyik kategóriába sorolható a kidobott rizs és egyéb köret nagy része? *Kérjük, válassza ki azt a kategóriát, amelyikből a legtöbb volt. Többet is választhat, ha több kategóriában ugyanannyi hulladék keletkezett.*

- ☐ Hiánytalan/bontatlan élelmiszerek: olyan kidobott élelmiszer, amely egyáltalán nem lett felhasználva (pl. egy teljes zacskó rizs)
- ☐ Felbontott/megkezdett élelmiszerek: olyan élelmiszerek, amelyekből valamennyi fogyott, de aztán kidobásra kerültek (pl. fél zacskó rizs)
- ☐ Ételmaradékok: tányéron, tálban, serpenyőben hagyott étel, ami kidobásra került.
- ☐ Tárolt ételmaradékok: későbbi fogyasztási céllal hűtőben vagy fagyasztóban tárolt ételmaradékok, amelyek végül a szemetesben végezték.

Question 18: Bab, lencse, csicseriborsó stb.
Mekkora mennyiségű hulladék keletkezett a háztartásában babból, lencséből, csicseriborsóból stb. az elmúlt hét folyamán?
Egy adag = fél merőkanál, kb. 50 gramm.

- Kevesebb, mint egy adag
- 1-2 adag
- 2-4 adag
- 4-6 adag
- Több, mint 6 adag

Question 19: Bab, lencse, csicseriborsó stb.
Melyik kategóriába sorolható a kidobott bab, lencse, csicseriborsó stb. nagy része? *Kérjük, válassza ki azt a kategóriát, amelyikből a legtöbb volt. Többet is választhat, ha több kategóriában ugyanannyi hulladék keletkezett.*

- ☐ Hiánytalan/bontatlan élelmiszerek: olyan kidobott élelmiszer, amely egyáltalán nem lett felhasználva (pl. bontatlan babkonzerv)
- ☐ Felbontott/megkezdett élelmiszerek: olyan élelmiszerek, amelyekből valamennyi fogyott, de aztán kidobásra kerültek (pl. fél konzerv bab)
- ☐ Ételmaradékok: tányéron, tálban, serpenyőben hagyott étel, ami kidobásra került.
- ☐ Tárolt ételmaradékok: későbbi fogyasztási céllal hűtőben vagy fagyasztóban tárolt ételmaradékok, amelyek végül a szemetesben végezték.

Question 20: Hús
Mekkora mennyiségű hulladék keletkezett a háztartásában húsból (kivéve: felvágott) az elmúlt hét folyamán?
Egy adag = egy csirkemell, egy steak stb. Kisebb darabok esetén (pl. darálthús), próbálja megbecsülni a mennyiségét egy normál adaghoz képest (pl. egy csomag fasírt két adagnak felel meg).

- Körülbelül fél adagnyi vagy kevesebb
- Körülbelül 1 adag
- 2-3 adag
- 4-5 adag
- Több, mint 5 adagnyi

Question 21: Hús
Melyik kategóriába sorolható a kidobott hús nagy része?
Kérjük, válassza ki azt a kategóriát, amelyikből a legtöbb volt. Többet is választhat, ha több kategóriában ugyanannyi hulladék keletkezett.

- ☐ Hiánytalan/bontatlan élelmiszerek: olyan kidobott élelmiszer, amely egyáltalán nem lett felhasználva (pl. egy csomag virsli)
- ☐ Felbontott/megkezdett élelmiszerek: olyan élelmiszerek, amelyekből valamennyi fogyott, de aztán kidobásra kerültek (pl. fél csomag virsli)
- ☐ Ételmaradékok: tányéron, tálban, serpenyőben hagyott étel, ami kidobásra került.
- ☐ Tárolt ételmaradékok: későbbi fogyasztási céllal hűtőben vagy fagyasztóban tárolt ételmaradékok, amelyek végül a szemetesben végezték.

Question 22: Húshelyettesítő
Mekkora mennyiségű hulladék keletkezett a háztartásában húshelyettesítőkből az elmúlt hét folyamán?
Egy adag = egy vegetariánius burger stb. Kisebb darabok esetén próbálja megbecsülni a mennyiségét egy normál adaghoz képest (pl. egy csomag vegetariánius fasírt két adagnak felel meg).

- Körülbelül fél adagnyi vagy kevesebb
- Körülbelül 1 adag
- 2-3 adag
- 4-5 adag
- Több, mint 5 adagnyi

Question 23: Húshelyettesítő
Melyik kategóriába sorolható a kidobott húshelyettesítő nagy része?
Kérjük, válassza ki azt a kategóriát, amelyikből a legtöbb volt. Többet is választhat, ha több kategóriában ugyanannyi hulladék keletkezett.

- ☐ Hiánytalan/bontatlan élelmiszerek: olyan kidobott élelmiszer, amely egyáltalán nem lett felhasználva (pl. vegetariánius burger csomag)
- ☐ Felbontott/megkezdett élelmiszerek: olyan élelmiszerek, amelyekből valamennyi fogyott, de aztán kidobásra kerültek (pl. fél vegetariánius burger csomag)
- ☐ Ételmaradékok: tányéron, tálban, serpenyőben hagyott étel, ami kidobásra került.
- ☐ Tárolt ételmaradékok: későbbi fogyasztási céllal hűtőben vagy fagyasztóban tárolt ételmaradékok, amelyek végül a szemetesben végezték.

Question 24: Hal
Mekkora mennyiségű hulladék keletkezett a háztartásában halból az elmúlt hét folyamán?
Egy adag = egy darab filézett haltörzs vagy lazac stb.
- Körülbelül fél adagnyi vagy kevesebb
- Körülbelül egy adag
- 2-3 adag
- 4-5 adag
- Több, mint 5 adag

Question 25: Hal
Melyik kategóriába sorolható a kidobott hal nagy része? *Kérjük, válassza ki azt a kategóriát, amelyikből a legtöbb volt. Többet is választhat, ha több kategóriában ugyanannyi hulladék keletkezett.*
- Hiánytalan/bontatlan élelmiszerek: olyan kidobott élelmiszer, amely egyáltalán nem lett felhasználva (pl. egy teljes csomag hal)
- Felbontott/megkezdett élelmiszerek: olyan élelmiszerek, amelyekből valamennyi fogyott, de aztán kidobásra kerültek (pl. fél csomag hal)
- Ételmaradékok: tányéron, tálban, serpenyőben hagyott étel, ami kidobásra került.
- Tárolt ételmaradékok: későbbi fogyasztási céllal hűtőben vagy fagyasztóban tárolt ételmaradékok, amelyek végül a szemetesben végezték.

Question 26: Kenyérfeltétek (felvágott, sajt, vaj, édes krém stb.
Mekkora mennyiségű hulladék keletkezett a háztartásában kenyérfeltétekből (felvágott, sajt, vaj, édes krém stb.) az elmúlt hét folyamán?
Egy adag: amit egy szelet kenyérre, zsömlébe, egy darab szendvicsbe használ fel.
- Körülbelül fél adag vagy kevesebb
- Körülbelül egy adag
- 2-3 adag
- 4-5 adag
- Több, mint 5 adag

Question 27: Kenyérfeltétek (felvágott, sajt, vaj, édes krém stb.
Melyik kategóriába sorolható a kidobott kenyérfeltét nagy része?
Kérjük, válassza ki azt a kategóriát, amelyikből a legtöbb volt. Többet is választhat, ha több kategóriában ugyanannyi hulladék keletkezett.
- Hiánytalan/bontatlan élelmiszerek: olyan kidobott élelmiszer, amely egyáltalán nem lett felhasználva (pl. egy teljes csomag felvágott)
- Felbontott/megkezdett élelmiszerek: olyan élelmiszerek, amelyekből valamennyi fogyott, de aztán kidobásra kerültek (pl. fél csomag felvágott)
- Ételmaradékok: tányéron, tálban, serpenyőben hagyott étel, ami kidobásra került.
- Tárolt ételmaradékok: későbbi fogyasztási céllal hűtőben vagy fagyasztóban tárolt ételmaradékok, amelyek végül a szemetesben végezték.

Question 28: Kenyér, zsemle, kifli stb
Mekkora mennyiségű hulladék keletkezett a háztartásában kenyérből, zsemléből, kifliből stb. az elmúlt hét folyamán?
Egy kifli, zsemle vagy szendvics nagyságrendileg egy szelet kenyérnek feleltethető meg.
- Kevesebb, mint egy szelet kenyér
- Egy vagy néhány szelet kenyér
- Körülbelül fél kiló kenyér
- Körülbelül egy kiló kenyér
- Több, mint egy kiló kenyér

Question 29: Kenyér, zsemle, kifli stb
Melyik kategóriába sorolható a kidobott kenyér, zsemle, kifli stb. nagy része? *Kérjük, válassza ki azt a kategóriát, amelyikből a legtöbb volt. Többet is választhat, ha több kategóriában ugyanannyi hulladék keletkezett.*
- Hiánytalan/bontatlan élelmiszerek: olyan kidobott élelmiszer, amely egyáltalán nem lett felhasználva (pl. egész kenyér)
- Felbontott/megkezdett élelmiszerek: olyan élelmiszerek, amelyekből valamennyi fogyott, de aztán kidobásra kerültek (pl. kenyérszeletek)
- Ételmaradékok: tányéron, tálban, serpenyőben hagyott étel, ami kidobásra került (pl. kenyérhéj)
- Tárolt ételmaradékok: későbbi fogyasztási céllal hűtőben vagy fagyasztóban tárolt ételmaradékok, amelyek végül a szemetesben végezték.

Question 30: Gabonapelyhek (müzli, zabpehely, zabkása, granola stb.
Mekkora mennyiségű hulladék keletkezett a háztartásában gabonapelyhekből (müzli, zabpehely, zabkása, granola stb.) az elmúlt hét folyamán?
Egy adag: amit egy tálkányi reggelihez felhasznál.

- Kevesebb, mint fél adagnyi
- Fél-másfél adagnyi
- Több adag (körülbelül fél csomag)
- Körülbelül egy egész csomaggal
- Több csomaggal

Question 31: Gabonapelyhek (müzli, zabpehely, zabkása, granola stb.
Melyik kategóriába sorolható a kidobott gabonapehely nagy része? *Kérjük, válassza ki azt a kategóriát, amelyikből a legtöbb volt. Többet is választhat, ha több kategóriában ugyanannyi hulladék keletkezett.*

- ☐ Hiánytalan/bontatlan élelmiszerek: olyan kidobott élelmiszer, amely egyáltalán nem lett felhasználva (pl. egy teljes csomag gabonapehely)
- ☐ Felbontott/megkezdett élelmiszerek: olyan élelmiszerek, amelyekből valamennyi fogyott, de aztán kidobásra kerültek (pl. fél csomag gabonapehely)
- ☐ Ételmaradékok: tányéron, tálban, serpenyőben hagyott étel, ami kidobásra került.
- ☐ Tárolt ételmaradékok: későbbi fogyasztási céllal hűtőben vagy fagyasztóban tárolt ételmaradékok, amelyek végül a szemetesben végezték.

Question 32: Joghurt, tejföl, túró stb.
Mekkora mennyiségű hulladék keletkezett a háztartásában joghurtból, tejfülből, túróból stb. az elmúlt hét folyamán?
Egy adag: egy kis tálka joghurt, tejföl, túró stb.

- Kevesebb, mint fél adagnyi
- Fél-másfél adagnyi
- Több adag (körülbelül fél liter)
- Körülbelül egy liter
- Több liternyi

Question 33: Joghurt, tejföl, túró stb.
Melyik kategóriába sorolható a kidobott joghurt, tejföl, túró stb. nagy része? *Kérjük, válassza ki azt a kategóriát, amelyikből a legtöbb volt. Többet is választhat, ha több kategóriában ugyanannyi hulladék keletkezett.*

- ☐ Hiánytalan/bontatlan élelmiszerek: olyan kidobott élelmiszer, amely egyáltalán nem lett felhasználva (pl. egy egész doboz joghurt)
- ☐ Felbontott/megkezdett élelmiszerek: olyan élelmiszerek, amelyekből valamennyi fogyott, de aztán kidobásra kerültek (pl. fél doboz joghurt)
- ☐ Ételmaradékok: tányéron, tálban, serpenyőben hagyott étel, ami kidobásra került.
- ☐ Tárolt ételmaradékok: későbbi fogyasztási céllal hűtőben vagy fagyasztóban tárolt ételmaradékok, amelyek végül a szemetesben végezték.

Question 34:
Sajt (tömbsajt, kockasajt, sajtkrém, kivéve: sajt kenyérfeltétként
Mekkora mennyiségű hulladék keletkezett a háztartásában különféle sajtokból (tömbsajt, kockasajt, sajtkrém, kivéve: sajt kenyérfeltétként) az elmúlt hét folyamán?
Egy adag: egy maréknyi sajt

- Kevesebb, mint egy adag
- Körülbelül egy adag
- 1-3 adag
- 4-5 adag t
- Több, mint 5 adag

Question 35: Sajt
Melyik kategóriába sorolható a kidobott sajtfélék nagy része? *Kérjük, válassza ki azt a kategóriát, amelyikből a legtöbb volt. Többet is választhat, ha több kategóriában ugyanannyi hulladék keletkezett.*

- ☐ Hiánytalan/bontatlan élelmiszerek: olyan kidobott élelmiszer, amely egyáltalán nem lett felhasználva (pl. felbontatlan csomag sajt)
- ☐ Felbontott/megkezdett élelmiszerek: olyan élelmiszerek, amelyekből valamennyi fogyott, de aztán kidobásra kerültek (pl. bontott csomag sajt)
- ☐ Ételmaradékok: tányéron, tálban, serpenyőben hagyott étel, ami kidobásra került.
- ☐ Tárolt ételmaradékok: későbbi fogyasztási céllal hűtőben vagy fagyasztóban tárolt ételmaradékok, amelyek végül a szemetesben végezték.

Question 36: Tojás
Mekkora mennyiségű hulladék keletkezett a háztartásában tojásból az elmúlt hét folyamán?

- Kevesebb, mint 1 tojás
- 1 tojás
- 2-3 tojás
- 4-5 tojás
- Több, mint 5 tojás

Question 37 : Tojás
Melyik kategóriába sorolható a kidobott tojás nagy része? *Kérjük, válassza ki azt a kategóriát, amelyikből a legtöbb volt. Többet is választhat, ha több kategóriában ugyanannyi hulladék keletkezett.*

- ☐ Hiánytalan/bontatlan élelmiszerek: olyan kidobott élelmiszer, amely egyáltalán nem lett felhasználva (pl. egész tojás)
- ☐ Felbontott/megkezdett élelmiszerek: olyan élelmiszerek, amelyekből valamennyi fogyott, de aztán kidobásra kerültek (pl. egy tojásfehérje)
- ☐ Ételmaradékok: tányéron, tálban, serpenyőben hagyott étel, ami kidobásra került.
- ☐ Tárolt ételmaradékok: későbbi fogyasztási céllal hűtőben vagy fagyasztóban tárolt ételmaradékok, amelyek végül a szemetesben végezték.

Question 38: Leves, főzelék
Mekkora mennyiségű hulladék keletkezett a háztartásában levesből és főzelékből az elmúlt hét folyamán?

- Kevesebb, mint fél merőkanálnyi
- Fél-másfél merőkanálnyi
- Több adag (körülbelül fél liter)
- Körülbelül egy liter
- Több, mint egy liter

Question 39: Leves, főzelék
Melyik kategóriába sorolható a kidobott levesek és főzelékek nagy része? *Kérjük, válassza ki azt a kategóriát, amelyikből a legtöbb volt. Többet is választhat, ha több kategóriában ugyanannyi hulladék keletkezett.*

- ☐ Hiánytalan/bontatlan élelmiszerek: olyan kidobott élelmiszer, amely egyáltalán nem lett felhasználva (pl. teljes csomag leves) – Otthon készített leves esetén nem releváns.
- ☐ Felbontott/megkezdett élelmiszerek: olyan élelmiszerek, amelyekből valamennyi fogyott, de aztán kidobásra kerültek (pl. fél csomag leves) – Otthon készített leves esetén nem releváns.
- ☐ Ételmaradékok: tányéron, tálban, serpenyőben hagyott étel, ami kidobásra került (pl. melegített kész leves vagy otthon főzött leves)
- ☐ Tárolt ételmaradékok: későbbi fogyasztási céllal hűtőben vagy fagyasztóban tárolt ételmaradékok, amelyek végül a szemetesben végezték.

Question 40: Szószok (ketchup, majonéz, mustár stb.
Mekkora mennyiségű hulladék keletkezett a háztartásában szószokból (ketchup, majonéz, mustár stb.) az elmúlt hét folyamán?
Egy evőkanál: 20 gramm.

- Kevesebb mint egy evőkanál
- 1-3 evőkanál
- Több, mint 3 evőkanál (Körülbelül fél tubus/üveg)
- Egy vagy több tubus/üveg
- Több, mint egy vagy több tubus/üveg

Question 41:Szószok (ketchup, majonéz, mustár stb.
Melyik kategóriába sorolható a kidobott szószok nagy része? *Kérjük, válassza ki azt a kategóriát, amelyikből a legtöbb volt. Többet is választhat, ha több kategóriában ugyanannyi hulladék keletkezett.*

- ☐ Hiánytalan/bontatlan élelmiszerek: olyan kidobott élelmiszer, amely egyáltalán nem lett felhasználva (pl. bontatlan tubus majonéz)
- ☐ Felbontott/megkezdett élelmiszerek: olyan élelmiszerek, amelyekből valamennyi fogyott, de aztán kidobásra kerültek (pl. fél üveg majonéz)
- ☐ Ételmaradékok: tányéron, tálban, serpenyőben hagyott étel, ami kidobásra került.
- ☐ Tárolt ételmaradékok: későbbi fogyasztási céllal hűtőben vagy fagyasztóban tárolt ételmaradékok, amelyek végül a szemetesben végezték.

Question 42: Édesség, sütemény, keksz, müzliszelet, csokoládé
Mekkora mennyiségű hulladék keletkezett a háztartásában édességekből (és sütemény, keksz, müzliszelet, csokoládé) az elmúlt hét folyamán?
Egy adag: egy maréknyi cukor, egy szelet csoki, egy süti stb.

- Körülbelül fél adagnyi vagy kevesebb
- Körülbelül 1 adag
- 2-3 adag
- 4-5 adag
- Több, mint 5 adag

Question 43: Édesség, sütemény, keksz, müzliszelet, csokoládé
Melyik kategóriába sorolható a kidobott édességek nagy része? *Kérjük, válassza ki azt a kategóriát, amelyikből a legtöbb volt. Többet is választhat, ha több kategóriában ugyanannyi hulladék keletkezett.*

- Hiánytalan/bontatlan élelmiszerek: olyan kidobott élelmiszer, amely egyáltalán nem lett felhasználva (pl. egy csomag keksz)
- Felbontott/megkezdett élelmiszerek: olyan élelmiszerek, amelyekből valamennyi fogyott, de aztán kidobásra kerültek (pl. fél csomag keksz)
- Ételmaradékok: tányéron, tálban, serpenyőben hagyott étel, ami kidobásra került.
- Tárolt ételmaradékok: későbbi fogyasztási céllal hűtőben vagy fagyasztóban tárolt ételmaradékok, amelyek végül a szemetesben végezték.

Question 44: Chipsek, ropik, magvak
Mekkora mennyiségű hulladék keletkezett a háztartásában chipsekből, ropikból és magvakból az elmúlt hét folyamán?
Egy adag: egy maréknyi mennyiség

- Körülbelül fél adagnyi vagy kevesebb
- Körülbelül 1 adag
- 2-3 adag
- 4-5 adag
- Több, mint 5 adag

Question 45: Chipsek, ropik, magvak
Melyik kategóriába sorolható a kidobott chipsek, ropik, magvak nagy része?
Kérjük, válassza ki azt a kategóriát, amelyikből a legtöbb volt. Többet is választhat, ha több kategóriában ugyanannyi hulladék keletkezett.

- Hiánytalan/bontatlan élelmiszerek: olyan kidobott élelmiszer, amely egyáltalán nem lett felhasználva (pl. egy zacskó chips)
- Felbontott/megkezdett élelmiszerek: olyan élelmiszerek, amelyekből valamennyi fogyott, de aztán kidobásra kerültek (pl. fél csomag chips)
- Ételmaradékok: tányéron, tálban, serpenyőben hagyott étel, ami kidobásra került.
- Tárolt ételmaradékok: későbbi fogyasztási céllal hűtőben vagy fagyasztóban tárolt ételmaradékok, amelyek végül a szemetesben végezték.

Question 46: Alkoholmentes italok
Mekkora mennyiségű hulladék keletkezett a háztartásában alkoholmentes italokból (tej, gyümölcslé, szénsavas üdítő, kivéve: víz, tea, kávé, higított szörp) az elmúlt hét folyamán?

- Kevesebb, mint fél pohár
- Fél-másfél pohár
- Több pohár (körülbelül fél liter)
- Körülbelül egy liter
- Több, mint egy liter

Question 47: Alkoholmentes italok
Melyik kategóriába sorolható a kidobott alkoholmentes italok nagy része?
Kérjük, válassza ki azt a kategóriát, amelyikből a legtöbb volt. Többet is választhat, ha több kategóriában ugyanannyi hulladék keletkezett.

- Hiánytalan/bontatlan élelmiszerek: olyan kidobott élelmiszer, amely egyáltalán nem lett felhasználva (pl. egy doboz tej)
- Felbontott/megkezdett élelmiszerek: olyan élelmiszerek, amelyekből valamennyi fogyott, de aztán kidobásra kerültek (pl. fél doboz tej)
- Maradék: pohárban megmaradt innivalók
- Tárolt maradékok: későbbi fogyasztási céllal hűtőben vagy fagyasztóban tárolt maradékok, amelyek végül a szemetesben végezték.

Question 48: Alkoholos italok

Mekkora mennyiségű hulladék keletkezett a háztartásában alkoholos italokból az elmúlt hét folyamán?

- o Kevesebb, mint fél pohár
- o Fél-másfél pohár
- o Több pohárral (körülbelül fél liter)
- o Körülbelül egy liter
- o Több, mint egy liter

Question 49: Alkoholos italok

Melyik kategóriába sorolható a kidobott alkoholos italok nagy része? *Kérjük, válassza ki azt a kategóriát, amelyikből a legtöbb volt. Többet is választhat, ha több kategóriában ugyanannyi hulladék keletkezett.*

- ☐ Hiánytalan/bontatlan élelmiszerek: olyan kidobott élelmiszer, amely egyáltalán nem lett felhasználva (pl. egy üveg bor)
- ☐ Felbontott/megkezdett élelmiszerek: olyan élelmiszerek, amelyekből valamennyi fogyott, de aztán kidobásra kerültek (pl. fél üveg bor)
- ☐ Maradék: pohárban megmaradt innivalók
- ☐ Tárolt maradékok: későbbi fogyasztási céllal hűtőben vagy fagyasztóban tárolt maradékok, amelyek végül a szemetesben végezték

Appendix E: Household Food Waste Questionnaire in Spanish

Information

La semana pasada usted recibió un email donde se le indicaba que prestara atención a los alimentos y bebidas que ha tirado. Este cuestionario es sobre esos alimentos.

Como recordatorio:

El cuestionario será sobre:

- La comida y bebida comprada en un supermercado, tienda especializada o comercio online, o que se ha cultivado en casa que se ha tirado
- Esto también incluye productos que se han estropeado, deteriorado o que han caducado o pasado la fecha de consumo.
- No importa si usted ha tirado estos alimentos o bebidas a la basura general, en el cubo de residuos orgánicos, en la compostadora o se ha dado a sus mascotas. Todo estaría incluido.

El cuestionario no será sobre:

- Huesos, pieles, semillas (partes no comestibles)
- Los alimentos y bebidas que tira cuando come en un restaurante o cafetería.

Questionnaire

Question 1: Por favor, marque las casillas de los productos que se tiraron en su hogar la semana pasada. En caso de platos completos marque las casillas de los ingredientes principales.

- ☐ Hortalizas frescas (apio, lechugas, calabacín, etcétera)
- ☐ Hortalizas no frescas (en bote de cristal / enlatadas / congeladas)
- ☐ Fruta fresca
- ☐ Fruta no fresca (en bote de cristal / enlatada / seca / congelada)
- ☐ Patatas
- ☐ Productos a base de patata (patatas fritas, patatas fritas congeladas, patatas precocidas, etcétera)
- ☐ Pasta
- ☐ Arroz y y otros granos (quinoa, cuscús, etcétera)
- ☐ Judías, lentejas, garbanzos, etcétera
- ☐ Carne
- ☐ Sustitutos de la carne (tofu, seitán, etcétera)
- ☐ Pescado
- ☐ Alimentos que se toman con pan (embutidos, queso en lonchas, mermeladas, crema de chocolate, etcétera)
- ☐ Pan
- ☐ Cereales (muesli, granola, avena, etcétera)
- ☐ Yogures, natillas, etcétera
- ☐ Quesos (cubitos de queso, queso francés, queso rayado, etcétera. Está excluido el queso de untar)
- ☐ Huevos
- ☐ Sopas o cremas
- ☐ Salsas (ketchup, mayonesa, salsa de cóctel, etcétera)
- ☐ Dulces (caramelos, barritas de cereales, barras de chocolate, etcétera) / galletas
- ☐ Aperitivos (snacks) y frutos secos
- ☐ Bebidas no alcohólicas (leche*, zumo, refrescos. Excluidas: agua, té, café)
- ☐ Bebidas alcohólicas
- ☐ No he tirado nada de comida o bebida

* *NOTE*: Due to progressive insights, creating a separate category for “Milk” can be considered, as this is a frequently disposed of food product. However, please keep in mind that this will change the set-up slightly, when comparing the results with the earlier applications of this survey.

Introduction to the next part of the questionnaire

Dividimos los residuos alimentarios en varias categorías, que se explican a continuación. Por favor, lea atentamente, ya que estas categorías se utilizarán en las preguntas siguientes.

1) <u>Alimentos completamente no utilizados</u>: alimentos que se tiran sin haber sido utilizados en absoluto. Por ejemplo: envases sin abrir, incluyendo partes no abiertas de multi-packs, manzanas podridas, puerros secos, panes enteros.
2) <u>Alimentos parcialmente utilizados:</u> los alimentos que se tiran después de haber sido parcialmente utilizados. Por ejemplo: unas pocas rebanadas de pan, la mitad de un paquete de filetes de carne, la mitad de una cebolla, la mitad de un brick de leche.
3) <u>Sobras de las comidas</u>: las sobras que se tiran después de haber sido dejadas en los platos, ollas o sartenes. Por ejemplo: arroz, puré de patata o pasta que se deja en el plato o en la sartén, bocadillos parcialmente comidos.
4) <u>Sobras almacenadas</u>: las sobras de comida que se tiran después de haber sido almacenadas en la nevera o congelador para ser comidas en un momento posterior. Por ejemplo: una porción de pasta congelada de la semana pasada.

Usted recibirá varias preguntas sobre diferentes tipos de alimentos y bebidas que haya tirado la semana pasada. Primero, le preguntaremos la cantidad de un cierto tipo de alimento que se tiró en su hogar la semana pasada. A continuación, le preguntaremos a qué categoría (completamente no utilizado, parcialmente utilizado, sobras de las comidas, sobras almacenadas) pertenecía la mayoría de este tipo de alimento en el momento de tirarse. Por favor, preste mucha atención a qué producto alimenticio se está refiriendo.

NOTE: The next questions are only shown if respondents indicated waste in the respective category in Question 1

Question 2: Hortalizas frescas
En su hogar, ¿qué cantidad de hortalizas frescas (apio, lechugas, calabacín, etcétera) se tiró la pasada semana?
Una cuchara de servir (cucharón) corresponde a 50 gramos. Como referencia esto equivale a medio puerro o cuatro champiñones.

- Menos de una cuchara de servir
- 1 a 2 cucharas de servir
- 2 a 4 cucharas de servir
- 4 a 6 cucharas de servir
- Más de 6 cucharas de servir

Question 3: Hortalizas frescas
¿A qué categoría pertenecían la mayoría de estas hortalizas frescas (apio, lechugas, calabacín, etcétera)? Por favor, marque la categoría más frecuente. Puede marcar más de una opción si se produjo la misma cantidad de varias categorías.

- ☐ Alimentos completamente no utilizados: alimentos que se tiran sin haber sido utilizados en absoluto. Por ejemplo: una cebolla.
- ☐ Alimentos parcialmente utilizados: los alimentos que se tiran después de haber sido parcialmente utilizados. Por ejemplo: la mitad de una cebolla.
- ☐ Sobras de las comidas: las sobras que se tiran después de haber sido dejadas en los platos, ollas o sartenes.
- ☐ Sobras almacenadas: las sobras de comida que se tiran después de haber sido almacenadas en la nevera o congelador para ser comidas en un momento posterior.

Question 4: Hortalizas no frescas
En su hogar, ¿qué cantidad de hortalizas no frescas (en bote / enlatadas / congeladas) se tiró la semana pasada?
Una cuchara de servir (cucharón) corresponde a 50 gramos. Como referencia esto equivale a medio puerro o cuatro champiñones.

- Menos de una cuchara de servir
- 1 a 2 cucharas de servir
- 2 a 4 cucharas de servir
- 4 a 6 cucharas de servir
- Más de 6 cucharas de servir

Question 5: Hortalizas no frescas
¿A qué categoría pertenecían la mayoría de estas hortalizas NO frescas?
Por favor, marque la categoría más frecuente. Puede marcar más de una opción si se produjo la misma cantidad de varias categorías.

- Alimentos completamente no utilizados: alimentos que se tiran sin haber sido utilizados en absoluto.
- Ejemplo, un paquete de espinacas congeladas / enlatadas no abierto
- Alimentos parcialmente utilizados: los alimentos que se tiran después de haber sido parcialmente utilizados. Por ejemplo: la mitad de un paquete de espinacas congeladas / enlatadas
- Sobras de las comidas: las sobras que se tiran después de haber sido dejadas en los platos, ollas o sartenes.
- Sobras almacenadas: las sobras de comida que se tiran después de haber sido almacenadas en la nevera o congelador para ser comidas en un momento posterior.

Question 6: Fruta fresca
En su hogar, ¿qué cantidad de frutas frescas se tiró la semana pasada?
Una manzana o plátano es una pieza de fruta. En el caso de frutas pequeñas, como fresas o uvas, un tazón pequeño se considera "una pieza".

- Aproximadamente un cuarto de una pieza de fruta o menos
- La mitad de una pieza de fruta
- Aproximadamente 1 pieza de fruta
- 2 a 4 piezas de fruta
- Más de 4 piezas de fruta

Question 7: Fruta fresca
¿A qué categoría pertenecían la mayoría de estas frutas frescas?
Por favor, marque la categoría más frecuente. Puede marcar más de una opción si se produjo la misma cantidad de varias categorías.

- Alimentos completamente no utilizados: alimentos que se tiran sin haber sido utilizados en absoluto. Por ejemplo: una manzana)
- Alimentos parcialmente utilizados: los alimentos que se tiran después de haber sido parcialmente utilizados. Por ejemplo: media manzana)
- Sobras de las comidas: las sobras que se tiran después de haber sido dejadas en los platos, ollas o sartenes. Por ejemplo: una manzana comida a medias o un poco de macedonia)
- Sobras almacenadas: las sobras de comida que se tiran después de haber sido almacenadas en la nevera o congelador para ser comidas en un momento posterior. Por ejemplo: una macedonia que se había guardado.

Question 8: Fruta no fresca
En su hogar, ¿qué cantidad de fruta NO fresca se tiró la semana pasada?
Una pera o melocotón de lata es una pieza de fruta. En el caso de frutas pequeñas, como arándanos o rodajas de piña, un tazón pequeño se considera "una pieza".

- Aproximadamente un cuarto de una pieza de fruta o menos
- La mitad de un pedazo de fruta
- Aproximadamente 1 pedazo de fruta
- 2 a 4 piezas de fruta
- Más de 4 piezas de fruta

Question 9: Fruta no fresca
¿A qué categoría pertenecían a mayoría de estas frutas NO frescas?
Por favor, marque la categoría más frecuente. Puede marcar más de una opción si se produjo la misma cantidad de varias categorías.

- Alimentos completamente no utilizados: alimentos que se tiran sin haber sido utilizados en absoluto. Por ejemplo: una lata de fruta sin abrir.
- Alimentos parcialmente utilizados: los alimentos que se tiran después de haber sido parcialmente utilizados. Por ejemplo: media lata de fruta
- Sobras de las comidas: las sobras que se tiran después de haber sido dejadas en los platos, ollas o sartenes.
- Sobras almacenadas: las sobras de comida que se tiran después de haber sido almacenadas en la nevera o congelador para ser comidas en un momento posterior.

Question 10: Patatas
En su hogar, ¿qué cantidad de patatas se tiró la semana pasada?
Una cuchara de servir (cucharón) corresponde a 50 gramos. Como referencia esto equivale a la mitad de una patata de tamaño medio.

- Menos de una cuchara de servir
- 1 a 2 cucharas de servir
- 2 a 4 cucharas de servir
- 4 a 6 cucharas de servir
- Más de 6 cucharas de servir

Question 11: Patatas
¿A qué categoría pertenecían la mayoría de estas patatas?
Por favor, marque la categoría más frecuente. Puede marcar más de una opción si se produjo la misma cantidad de varias categorías.

- ☐ Alimentos completamente no utilizados: alimentos que se tiran sin haber sido utilizados en absoluto. Por ejemplo: un paquete de patatas completo)
- ☐ Alimentos parcialmente utilizados: los alimentos que se tiran después de haber sido parcialmente utilizados. Por ejemplo: medio paquete de patatas)
- ☐ Sobras de las comidas: las sobras que se tiran después de haber sido dejadas en los platos, ollas o sartenes. Por ejemplo: puré de patatas.
- ☐ Sobras almacenadas: las sobras de comida que se tiran después de haber sido almacenadas en la nevera o congelador para ser comidas en un momento posterior. Por ejemplo puré de patatas después de haber sido guardado.

Question 12: Productos a base de patata
En su hogar, ¿qué cantidad de productos a base de patata se tiró la semana pasada?

- Menos de 10 patatas fritas / patatas baby / piezas
- 10 a 25 patatas fritas / patatas baby / piezas
- Más de 25 patatas fritas / patatas baby / piezas (aproximadamente la mitad de un paquete de 500 gramos)
- Un paquete completo (750 gramos) patatas fritas / patatas baby/ piezas
- Más de un paquete (750 gramos) de patatas / patatas baby / piezas

Question 13: Productos a base de patata
¿A qué categoría pertenecían la mayoría de estos productos a base de patata?
Por favor, marque la categoría más frecuente. Puede marcar más de una opción si se produjo la misma cantidad de varias categorías.

- ☐ Alimentos completamente no utilizados: alimentos que se tiran sin haber sido utilizados en absoluto. Por ejemplo: un paquete de patatas fritas completo.
- ☐ Alimentos parcialmente utilizados: los alimentos que se tiran después de haber sido parcialmente utilizados. Por ejemplo: medio paquete de patatas fritas.
- ☐ Sobras de las comidas: las sobras que se tiran después de haber sido dejadas en los platos, ollas o sartenes.
- ☐ Sobras almacenadas: las sobras de comida que se tiran después de haber sido almacenadas en la nevera o congelador para ser comidas en un momento posterior.

Question 14: Pasta
En su hogar, ¿qué cantidad de pasta se tiró la semana pasada?
Una cuchara de servir (cucharón) corresponde a 50 gramos.

- Menos de una cuchara de servir
- 1 a 2 cucharas de servir
- 2 a 4 cucharas de servir
- 4 a 6 cucharas de servir
- Más de 6 cucharas de servir

Question 15: Pasta
¿A qué categoría pertenecía la mayoría de esta pasta?
Por favor, marque la categoría más frecuente. Puede marcar más de una opción si se produjo la misma cantidad de varias categorías.

- Alimentos completamente no utilizados: alimentos que se tiran sin haber sido utilizados en absoluto. Por ejemplo: un paquete de pasta completo.
- Alimentos parcialmente utilizados: los alimentos que se tiran después de haber sido parcialmente utilizados. Por ejemplo: medio paquete de pasta.
- Sobras de las comidas: las sobras que se tiran después de haber sido dejadas en los platos, ollas o sartenes.
- Sobras almacenadas: las sobras de comida que se tiran después de haber sido almacenadas en la nevera o congelador para ser comidas en un momento posterior.

Question 16: Arroz y otros granos
En su hogar, ¿qué cantidad de arroz y otros granos (quinoa, cuscús, etcétera) se tiró la semana pasada?
Una cuchara de servir (cucharón) corresponde a 50 gramos.

- Menos de una cuchara de servir
- 1 a 2 cucharas de servir
- 2 a 4 cucharas de servir
- 4 a 6 cucharas de servir
- Más de 6 cucharas de servir

Question 17: Arroz y y otros granos
¿A qué categoría pertenecían la mayoría de arroz y otros granos (quinoa, cuscús, etcétera)?
Por favor, marque la categoría más frecuente. Puede marcar más de una opción si se produjo la misma cantidad de varias categorías

- Alimentos completamente no utilizados: alimentos que se tiran sin haber sido utilizados en absoluto. Por ejemplo: un paquete completo de arroz.
- Alimentos parcialmente utilizados: los alimentos que se tiran después de haber sido parcialmente utilizados. Por ejemplo: medio paquete de arroz.
- Sobras de las comidas: las sobras que se tiran después de haber sido dejadas en los platos, ollas o sartenes.
- Sobras almacenadas: las sobras de comida que se tiran después de haber sido almacenadas en la nevera o congelador para ser comidas en un momento posterior.

Question 18: Judías, lentejas, garbanzos, etcétera
En su hogar, ¿qué cantidad de judías, lentejas, garbanzos, etcéra se tiró la semana pasada?
Una cuchara de servir (cucharón) corresponde a 50 gramos.

- Menos de una cuchara de servir
- 1 a 2 cucharas de servir
- 2 a 4 cucharas de servir
- 4 a 6 cucharas de servir
- Más de 6 cucharas de servir

Question 19: Judías, lentejas, garbanzos, etcétera
¿A qué categoría pertenecían la mayoría judías, lentejas, garbanzos, etcéra?
Por favor, marque la categoría más frecuente. Puede marcar más de una opción si se produjo la misma cantidad de varias categorías.

- Alimentos completamente no utilizados: alimentos que se tiran sin haber sido utilizados en absoluto. Por ejemplo, un tarro de judías sin abrir.
- Alimentos parcialmente utilizados: los alimentos que se tiran después de haber sido parcialmente utilizados. Por ejemplo, medio tarro de garbanzos.
- Sobras de las comidas: las sobras que se tiran después de haber sido dejadas en los platos, ollas o sartenes.
- Sobras almacenadas: las sobras de comida que se tiran después de haber sido almacenadas en la nevera o congelador para ser comidas en un momento posterior.

Question 20: Carne
En su hogar, ¿qué cantidad de carne se tiró la semana pasada?
Una porción se refiere a una pechuga de pollo, un filete de carne, etcétera. En el caso de piezas más pequeñas, como la carne picada, trate de estimarla en pedazos enteros de carne.

- Aproximadamente la mitad de una porción o menos
- Aproximadamente 1 porción
- 2 a 3 porciones
- 4 a 5 porciones
- Más de 5 porciones

Question 21: Carne
¿A qué categoría pertenecía la mayoría de esta carne?
Por favor, marque la categoría más frecuente. Puede marcar más de una opción si se produjo la misma cantidad de varias categorías.

- [ ] Alimentos completamente no utilizados: alimentos que se tiran sin haber sido utilizados en absoluto. Por ejemplo: un paquete de salchichas.
- [ ] Alimentos parcialmente utilizados: los alimentos que se tiran después de haber sido parcialmente utilizados. Por ejemplo: medio paquete de salchichas.
- [ ] Sobras de las comidas: las sobras que se tiran después de haber sido dejadas en los platos, ollas o sartenes.
- [ ] Sobras almacenadas: las sobras de comida que se tiran después de haber sido almacenadas en la nevera o congelador para ser comidas en un momento posterior.

Question 22: Sustitutos de la carne
En su hogar, ¿qué cantidad de sustitutos de carne se tiró la semana pasada?
Una porción se refiere a una hamburguesa vegetariana o un filete de seitán.

- o Aproximadamente la mitad de una porción o menos
- o Aproximadamente 1 porción
- o 2 a 3 porciones
- o 4 a 5 porciones
- o Más de 5 porciones

Question 23: Sustitutos de la carne
¿A qué categoría pertenecían la mayoría de estos sustitutos de la carne?
Por favor, marque la categoría más frecuente. Puede marcar más de una opción si se produjo la misma cantidad de varias categorías.

- [ ] Alimentos completamente no utilizados: alimentos que se tiran sin haber sido utilizados en absoluto. Por ejemplo: un paquete de hamburguesas vegetarianas.
- [ ] Alimentos parcialmente utilizados: los alimentos que se tiran después de haber sido parcialmente utilizados. Por ejemplo: medio filete de seitán
- [ ] Sobras de las comidas: las sobras que se tiran después de haber sido dejadas en los platos, ollas o sartenes.
- [ ] Sobras almacenadas: las sobras de comida que se tiran después de haber sido almacenadas en la nevera o congelador para ser comidas en un momento posterior.

Question 24: Pescado
En su hogar, ¿qué cantidad de pescado se tiró la semana pasada?
Una porción se refiere a un filete de pescado entero, una pieza de salmón, etcétera.

- o Aproximadamente la mitad de una porción o menos
- o Aproximadamente 1 porción completa
- o 2 a 3 porciones
- o 4 a 5 porciones
- o Más de 5 porciones

Question 25: Pescado
¿A qué categoría pertenecía la mayoría de este pescado?
Por favor, marque la categoría más frecuente. Puede marcar más de una opción si se produjo la misma cantidad de varias categorías.

- [ ] Alimentos completamente no utilizados: alimentos que se tiran sin haber sido utilizados en absoluto. Por ejemplo: un paquete de pescado entero.
- [ ] Alimentos parcialmente utilizados: los alimentos que se tiran después de haber sido parcialmente utilizados. Por ejemplo: medio paquete de pescado.
- [ ] Sobras de las comidas: las sobras que se tiran después de haber sido dejadas en los platos, ollas o sartenes.
- [ ] Sobras almacenadas: las sobras de comida que se tiran después de haber sido almacenadas en la nevera o congelador para ser comidas en un momento posterior.

Question 26: Alimentos que se toman con pan
En su hogar, ¿qué cantidad de alimentos que se toman con pan (embutidos, queso en lonchas, mermeladas, crema de chocolate, etcétera) se tiró la semana pasada?
Una porción es lo que se utiliza en una rebanada de pan / sándwich / porción de baguette.

- Aproximadamente la mitad de una porción o menos
- Aproximadamente 1 porción completa
- 2 a 3 porciones
- 4 a 5 porciones
- Más de 5 porciones

Question 27: Alimentos que se toman con pan
¿A qué categoría pertenecían la mayoría de estos Alimentos que se toman con pan (embutidos, queso en lonchas, mermeladas, crema de chocolate, etcétera)?
Por favor, marque la categoría más frecuente. Puede marcar más de una opción si se produjo la misma cantidad de varias categorías.

- Alimentos completamente no utilizados: alimentos que se tiran sin haber sido utilizados en absoluto. Por ejemplo: un paquete entero de lonchas de embutido.
- Alimentos parcialmente utilizados: los alimentos que se tiran después de haber sido parcialmente utilizados. Por ejemplo: medio paquete de lonchas de embutido.
- Sobras de las comidas: las sobras que se tiran después de haber sido dejadas en los platos, ollas o sartenes.
- Sobras almacenadas: las sobras de comida que se tiran después de haber sido almacenadas en la nevera o congelador para ser comidas en un momento posterior.

Question 28: Pan
En su hogar, ¿qué cantidad de pan se tiró la semana pasada?
Un bollo, una porción de baguette o sándwich es similar a una rebanada de pan.

- Menos de una rebanada de pan
- 1 o varias rebanadas de pan
- Aproximadamente la mitad de una hogaza de pan (una hogaza es el equivalente a dos barras de medio)
- Aproximadamente 1 hogaza de pan (una hogaza es el equivalente a dos barras de medio)
- Más de una hogaza de pan

Question 29: Pan
¿A qué categoría pertenecía la mayoría de este pan?
Por favor, marque la categoría más frecuente. Puede marcar más de una opción si se produjo la misma cantidad de varias categorías.

- Alimentos completamente no utilizados: alimentos que se tiran sin haber sido utilizados en absoluto. Por ejemplo, una barra de pan entera.
- Alimentos parcialmente utilizados: los alimentos que se tiran después de haber sido parcialmente utilizados. Por ejemplo: unas rebanadas de pan.
- Sobras de las comidas: las sobras que se tiran después de haber sido dejadas en los platos, ollas o sartenes.
- Sobras almacenadas: las sobras de comida que se tiran después de haber sido almacenadas en la nevera o congelador para ser comidas en un momento posterior.

Question 30: Cereales
En su hogar, ¿qué cantidad de cereales (muesli, granola, avena, etc.) se tiró la semana pasada?
Una porción es la cantidad de cereales usados para un tazón de desayuno.

- Menos de la mitad de una porción
- De una mitad de porción a una porción y media
- Varias porciones (aproximadamente la mitad de un paquete)
- Aproximadamente un paquete completo
- Varios paquetes

Question 31: Cereales
¿A qué categoría pertenecían la mayoría de estos cereales?
Por favor, marque la categoría más frecuente. Puede marcar más de una opción si se produjo la misma cantidad de varias categorías.

- Alimentos completamente no utilizados: alimentos que se tiran sin haber sido utilizados en absoluto. (Por ejemplo, un paquete completo de cereales)
- Alimentos parcialmente utilizados: los alimentos que se tiran después de haber sido parcialmente utilizados. (por ejemplo, medio paquete de cereales)
- Sobras de las comidas: las sobras que se tiran después de haber sido dejadas en los platos, ollas o sartenes.
- Sobras almacenadas: las sobras de comida que se tiran después de haber sido almacenadas en la nevera o congelador para ser comidas en un momento posterior.

Question 32: Yogures
En su hogar, ¿qué cantidad de yogur, natillas, etcétera se tiró la semana pasada?
Una porción es un tazón pequeño con yogur / natillas / etcétera.

- Menos de la mitad de la porción
- De una mitad a una porción y media
- Varias porciones (aproximadamente un brick de medio litro)
- Aproximadamente 1 brick de litro entero
- Varios bricks de litro

Question 33: Yogures
¿A qué categoría pertenecían la mayoría de estos yogures, natillas, etc.?
Por favor, marque la categoría más frecuente. Puede marcar más de una opción si se produjo la misma cantidad de varias categorías.

- Alimentos completamente no utilizados: alimentos que se tiran sin haber sido utilizados en absoluto. Por ejemplo: un pack completo de yogures.
- Alimentos parcialmente utilizados: los alimentos que se tiran después de haber sido parcialmente utilizados. Por ejemplo: medio paquete de yogures.
- Sobras de las comidas: las sobras que se tiran después de haber sido dejadas en los platos, ollas o sartenes.
- Sobras almacenadas: las sobras de comida que se tiran después de haber sido almacenadas en la nevera o congelador para ser comidas en un momento posterior.

Question 34: Quesos
En su hogar, ¿qué cantidad de queso (lonchas de queso, queso francés, queso para espolvorear, etcétera (excluyendo el queso de untar) se tiró la semana pasada?
Una porción equivale a un dado o triángulo de queso.
Un puñado de queso se puede ver como un dado de queso.

- Menos de una porción
- Aproximadamente una porción
- 1 a 3 porciones
- 4 a 5 porciones
- Más de 5 porciones

NOTE with question 34: Due to progressive insights, increasing the measure options can be considered, as 5 cheese dices is low compared to the potential maximum gram of waste of the other categories. However, please keep in mind that this will change the set-up slightly, when comparing the results with the earlier applications of this survey.

Question 35: Quesos
¿A qué categoría pertenecía la mayoría de este queso?
Por favor, marque la categoría más frecuente. Puede marcar más de una opción si se produjo la misma cantidad de varias categorías.

- Alimentos completamente no utilizados: alimentos que se tiran sin haber sido utilizados en absoluto. Por ejemplo, un queso entero.
- Alimentos parcialmente utilizados: los alimentos que se tiran después de haber sido parcialmente utilizados. Por ejemplo: medio queso.
- Sobras de las comidas: las sobras que se tiran después de haber sido dejadas en los platos, ollas o sartenes.
- Sobras almacenadas: las sobras de comida que se tiran después de haber sido almacenadas en la nevera o congelador para ser comidas en un momento posterior.

Question 36: Huevos
En su hogar, ¿cuántos huevos se tiraron la semana pasada?
- Menos de 1 huevo
- 1 huevo
- 2 a 3 huevos
- 4 a 5 huevos
- Más de 5 huevos

Question 37: Huevos
¿A qué categoría pertenecían la mayoría de estos huevos?
Por favor, marque la categoría más frecuente. Puede marcar más de una opción si se produjo la misma cantidad de varias categorías.
- ☐ Alimentos completamente no utilizados: alimentos que se tiran sin haber sido utilizados en absoluto. Por ejemplo, un huevo entero.
- ☐ Alimentos parcialmente utilizados: los alimentos que se tiran después de haber sido parcialmente utilizados. Por ejemplo: la clara de un huevo.
- ☐ Sobras de las comidas: las sobras que se tiran después de haber sido dejadas en los platos, ollas o sartenes.
- ☐ Sobras almacenadas: las sobras de comida que se tiran después de haber sido almacenadas en la nevera o congelador para ser comidas en un momento posterior.

Question 38: Sopas o cremas
En su hogar, ¿qué cantidad de sopa se tiró la semana pasada?
- Menos de la mitad de un cucharón
- De medio cucharón a un cucharón y medio
- Varios cucharones (aproximadamente medio litro)
- Aproximadamente 1 litro
- Más de 1 litro

Question 39: Sopas o cremas
¿A qué categoría pertenecían la mayoría de estas sopas?
Por favor, marque la categoría más frecuente. Puede marcar más de una opción si se produjo la misma cantidad de varias categorías.
- ☐ Alimentos completamente no utilizados: alimentos que se tiran sin haber sido utilizados en absoluto. Por ejemplo: un paquete de sopa entero. (No aplica en caso de sopa casera)
- ☐ Alimentos parcialmente utilizados: los alimentos que se tiran después de haber sido parcialmente utilizados. Por ejemplo: medio paquete de sopa. (No aplica en caso de sopa casera)
- ☐ Sobras de las comidas: las sobras que se tiran después de haber sido dejadas en los platos, ollas o sartenes. (Sopa calentada del brick o cocinada casera)
- ☐ Sobras almacenadas: las sobras de comida que se tiran después de haber sido almacenadas en la nevera o congelador para ser comidas en un momento posterior.

Question 40: Salsas
En su hogar, ¿qué cantidad de salsas (ketchup, mayonesa, salsa de cóctel, etcétera) se tiró la semana pasada?
Una cuchara sopera equivale a 20 gramos.
- Menos de una cuchara sopera
- 1 a 3 cucharas de soperas
- Varias cucharas soperas (aproximadamente medio bote / botella)
- Aproximadamente un bote / botella
- Más de un bote / botella

Question 41: Salsas
¿A qué categoría pertenecían la mayoría de estas salsas?
Por favor, marque la categoría más frecuente. Puede marcar más de una opción si se produjo la misma cantidad de varias categorías.

- ☐ Alimentos completamente no utilizados: alimentos que se tiran sin haber sido utilizados en absoluto. (Por ejemplo, un bote de salsa entero)
- ☐ Alimentos parcialmente utilizados: los alimentos que se tiran después de haber sido parcialmente utilizados. Por ejemplo, medio bote de salsa.
- ☐ Sobras de las comidas: las sobras que se tiran después de haber sido dejadas en los platos, ollas o sartenes.
- ☐ Sobras almacenadas: las sobras de comida que se tiran después de haber sido almacenadas en la nevera o congelador para ser comidas en un momento posterior.

Question 42; Dulces
En su hogar, ¿qué cantidad de dulces (caramelos, barras de chocolate, etcétera) / galletas se tiró la semana pasada?
Una porción es un puñado de caramelos, una pequeña barra de chocolate, una galleta, etcétera.

- Aproximadamente la mitad de una porción o menos
- Aproximadamente 1 porción
- 2 a 3 porciones
- 4 a 5 porciones
- Más de 5 porciones

Question 43: Dulces
¿A qué categoría pertenecían la mayoría de estos dulces?
Por favor, marque la categoría más frecuente. Puede marcar más de una opción si se produjo la misma cantidad de varias categorías.

- Alimentos completamente no utilizados: alimentos que se tiran sin haber sido utilizados en absoluto. Por ejemplo: un paquete de galletas.
- Alimentos parcialmente utilizados: los alimentos que se tiran después de haber sido parcialmente utilizados. Por ejemplo, medio paquete de galletas.
- Sobras de las comidas: las sobras que se tiran después de haber sido dejadas en los platos, ollas o sartenes.
- Sobras almacenadas: las sobras de comida que se tiran después de haber sido almacenadas en la nevera o congelador para ser comidas en un momento posterior.

Question 44: Aperitivos (snacks)
En su hogar, ¿qué cantidad de aperitivos (snacks) y frutos secos se tiraron la semana pasada? Una porción es un puñado de aperitivos (snacks) y frutos secos.
Una porción es un puñado de snacks o frutos secos

- Aproximadamente la mitad de una porción o menos
- Aproximadamente 1 porción
- 2 a 3 porciones
- 4 a 5 porciones
- Más de 5 porciones

Question 45: Aperitivos (snacks)
¿A qué categoría pertenecían la mayoría de estos aperitivos (snacks) y frutos secos?
Por favor, marque la categoría más frecuente. Puede marcar más de una opción si se produjo la misma cantidad de varias categorías.

- Alimentos completamente no utilizados: alimentos que se tiran sin haber sido utilizados en absoluto. Por ejemplo, una bolsa de snacks.
- Alimentos parcialmente utilizados: los alimentos que se tiran después de haber sido parcialmente utilizados. Por ejemplo: media bolsa de snacks.
- Sobras de las comidas: las sobras que se tiran después de haber sido dejadas en los platos, ollas o sartenes.
- Sobras almacenadas: las sobras de comida que se tiran después de haber sido almacenadas en la nevera o congelador para ser comidas en un momento posterior.

Question 46: Bebidas no alcohólicas
En su hogar, ¿qué cantidad de bebidas no alcohólicas (leche, zumo, refrescos, etc. Excluido: agua, té, café, siropes) se tiró la semana pasada?

- Menos de medio vaso
- De medio vaso a un vaso y medio
- Varios vasos (aproximadamente medio litro)
- Aproximadamente 1 litro
- Más de 1 litro

Question 47: Bebidas no alcohólicas
¿A qué categoría pertenecían la mayoría de las bebidas no alcohólicas?
Por favor, marque la categoría más frecuente. Puede marcar más de una opción si se produjo la misma cantidad de varias categorías.

- Alimentos completamente no utilizados: bebidas que se tiran sin haber sido utilizados en absoluto. Por ejemplo: un brick de leche.
- Alimentos parcialmente utilizados: bebidas que se tiran después de haber sido parcialmente utilizados. Por ejemplo: medio brick de leche.
- Sobras de las comidas: bebida que queda en el vaso
- Sobras almacenadas: las sobras de bebidas que se tiran después de haber sido almacenadas en la nevera o congelador para ser consumidas en un momento posterior.

Question 48: Bebidas alcohólicas
En su hogar, ¿qué cantidad de bebidas alcohólicas se tiró la semana pasada?

- o Menos de la mitad de un vaso de cerveza o vino
- o De medio vaso de cerveza o vino a un vaso y medio
- o Varios vasos de cerveza o vino (aproximadamente medio litro)
- o Aproximadamente 1 litro
- o Más de 1 litro

Question 49: Bebidas alcohólicas
¿A qué categoría pertenecían la mayoría de estas bebidas alcohólicas?
Por favor, marque la categoría más frecuente. Puede marcar más de una opción si se produjo la misma cantidad de varias categorías.

- ☐ Alimentos completamente no utilizados: bebidas que se tiran sin haber sido utilizadas en absoluto. Por ejemplo: una botella de vino.
- ☐ Alimentos parcialmente utilizados: bebidas que se tiran después de haber sido parcialmente utilizadas. Por ejemplo: media botella de vino.
- ☐ Sobras de las comidas: bebida que queda en el vaso
- ☐ Sobras almacenadas: las sobras de bebidas que se tiran después de haber sido almacenadas en la nevera o congelador para ser consumidas en un momento posterior.

Appendix F: Amount of grams per unit and amount of grams per answer option of the Household Food Waste Questionnaire

Category	Unit	Estimated grams
Fresh vegetables	Serving spoon	50
Non-fresh vegetables	Serving spoon	50
Fresh fruit	Piece	100
Non-fresh fruit	Piece	80
Potatoes	Serving spoon	60
Potato products	10 fries	50
Pasta	Serving spoon	50
Rice	Serving spoon	60
Meat	Portion	150
Fish	Portion	150
Sandwich filling	Portion	20
Bread	Slice	35
Bread	Whole bread	800
Cereals	Portion	40
Cereals	Pack	500
Yoghurt et cetera	Portion	150
Yoghurt et cetera	Pack	1000
Cheese	Cube	10
Eggs	Egg	60
Soups	Ladle	150
Soups	Litre	1000
Sauces	Spoon	20
Sauces	Bottle	450
Candy	Portion	20
Crisps / nuts	Portion	20
Non-alcoholic beverage	Glass	250
Non-alcoholic beverage	Litre	1000
Alcoholic beverage	Beer glass	300
Alcoholic beverage	Litre	1000

Product category	Answer option 1	Answer option 2	Answer option 3	Answer option 4	Answer option 5
Fresh vegetables and salads	25	75	150	250	350
Non-fresh vegetables	25	75	150	250	350
Fresh fruit	25	50	100	300	500
Non-fresh fruit	20	40	80	240	400
Potatoes	25	75	150	250	350
Potato products	25	88	375	750	1125
Pasta	25	75	150	250	350
Rice and remaining grains	25	75	150	250	350
Beans, lentils, chickpeas, et cetera	25	75	150	250	350
Meat	75	150	375	675	900
Meat substitute	45	90	225	405	540
Fish	75	150	375	675	900
Bread toppings	10	20	50	90	120
Bread	18	35	400	800	1200
Cereals	10	40	250	500	1000
Yoghurt, custard, et cetera	38	150	500	1000	2000
Cheese	5	10	20	45	60
Eggs	30	60	150	270	360
Soups / curry	38	150	500	1000	1500
Sauce	10	30	90	225	675
Candy / cookies / bars	10	20	50	90	120
Crisps / nuts	10	20	50	90	120
Non-alcoholic beverages	68	250	500	1000	1500
Alcoholic beverages	75	300	500	1000	1500

Estimates are based on online information available from the Dutch food and nutrition centre (voedingscentrum.nl), a major Dutch retailer (ah.nl), two platforms dedicated to weight loss / healthy eating (wijvallenaf.nl and smakelijketenzonderzout), supplemented with own measurements.
